# Supplementary material for: Transcriptome-wide and stratified genomic structural equation modeling identify neurobiological pathways shared across diverse cognitive traits
Source: Nat Commun. 2022 Oct 21;13:6280. doi: 10.1038/s41467-022-33724-9 (PMC9586980; doi:10.1038/s41467-022-33724-9)
Supplement: Supplementary file 1 — Supplementary Information [file 41467_2022_33724_MOESM1_ESM.pdf]

Online Supplement

Transcriptome-wide and Stratified Genomic Structural Equation Modeling Identify Neurobiological Pathways Underlying General and Specific Cognitive Functions

Table of Contents

Supplementary Figure 1. T-SEM Models....3
Supplementary Figure 2a. Histograms of T-SEM Simulation Results for ZSCAN9....4
Supplementary Figure 2b. Histograms of T-SEM Simulation Results for ZNF749....5
Supplementary Figure 3a. Histograms of Q_{Gene} Simulation Results for ZSCAN9....6
Supplementary Figure 3b. Histograms of Q_{Gene} Simulation Results for ZNF749....7
Supplementary Figure 4a. QQ-plot of T-SEM Simulation Results for ZCAN9....8
Supplementary Figure 4b. QQ-plot of T-SEM Simulation Results for ZNF749....9
Supplementary Figure 5a. QQ-plot of Q_{Gene} Simulation Results for ZSCAN9....10
Supplementary Figure 5b. QQ-plot of Q_{Gene} Simulation Results for ZNF749....11
Supplementary Figure 6. Scatterplots of TWAS versus T-SEM Simulation Results....12
Supplementary Figure 7. QQ-plot of SNP-level Simulation Results....13
Supplementary Figure 8. Simulations Incorporating Sampling Variation in b_{eQTL} Estimates....14
Supplementary Figure 9a. Regional Association Plot for Locus 1 from g-factor Conditional Analyses. The....15
Supplementary Figure 9b. Regional Association Plot for Locus 2 from g-factor Conditional Analyses....16
Supplementary Figure 9c. Regional Association Plot for Locus 3 from g-factor Conditional Analyses....17
Supplementary Figure 9d. Regional Association Plot for Locus 4 from g-factor Conditional Analyses....18
Supplementary Figure 9e. Regional Association Plot for Locus 6 from g-factor Conditional Analyses....19
Supplementary Figure 9f. Regional Association Plot for Locus 7 from g-factor Conditional Analyses....20
Supplementary Figure 9g. Regional Association Plot for Locus 8 from g-factor Conditional Analyses....21
Supplementary Figure 9h. Regional Association Plot for Locus 9 from g-factor Conditional Analyses....22
Supplementary Figure 9i. Regional Association Plot for Locus 10 from g-factor Conditional Analyses....23
Supplementary Figure 9k. Regional Association Plot for Locus 12 from g-factor Conditional Analyses....25
Supplementary Figure 9l. Regional Association Plot for Locus 13 from g-factor Conditional Analyses....26
Supplementary Figure 9m. Regional Association Plot for Locus 14 from g-factor Conditional Analyses....27
Supplementary Figure 9n. Regional Association Plot for Locus 15 from g-factor Conditional Analyses....28
Supplementary Figure 9o. Regional Association Plot for Locus 16 from g-factor Conditional Analyses....29
Supplementary Figure 9p. Regional Association Plot for Locus 17 from g-factor Conditional Analyses....30
Supplementary Figure 9q. Regional Association Plot for Locus 18 from g-factor Conditional Analyses....31
Supplementary Figure 10. g-factor Gene Co-expression Network....32
Supplementary Figure 11. Q_{Gene} Gene Co-expression Network....33

Supplementary Figure 12. Scatterplot of 17q21.31 Q_{Gene} hits. 34
Supplementary Figure 13. Mean chi-square across Tissues. 35
Supplementary Figure 14. TWAS vs T-SEM of *g*-factor. 36
Supplementary Figure 15. Factor Models estimated using Genome-wide, Zero-order Matrix....37
Supplementary Figure 16a. Enrichment of Baseline Annotations for g-factor. 38
Supplementary Figure 16b. Enrichment of MAF Annotations for g-factor. 39
Supplementary Figure 16c. Enrichment of Gene Expression Annotations for g-factor. 40
Supplementary Figure 16d. Enrichment of Histone Mark Annotations for g-factor. 41
Supplementary Figure 16e. Enrichment of PI x Brain Cell Annotations for g-factor. 42
Supplementary Figure 17. Scatter plots for *g* enrichment fit to stratified covariance and correlation matrices. 43
Supplementary Figure 18. Scatterplots of residual and genetic *g* enrichment....44
Supplementary Figure 19a. Enrichment for Residual Variance in Reaction Time. 45
Supplementary Figure 19b. Enrichment for Residual Variance in Verbal Numerical Reasoning. 46
Supplementary Figure 19c. Enrichment for Residual Variance in the Memory Pairs-matching Test. 47
Supplementary Figure 20a. Enrichment of Baseline Annotations for covariance between the *g*-factor and psychotic disorders factor. 48
Supplementary Figure 20b. Enrichment of MAF Annotations for covariance between the *g*-factor and psychotic disorders factor. 49
Supplementary Figure 20c. Enrichment of Gene Expression Annotations for covariance between the *g*-factor and psychotic disorders factor. 50
Supplementary Figure 20d. Enrichment of Histone Mark Annotations for covariance between the *g*-factor and psychotic disorders factor 51
Supplementary Figure 20e. Enrichment of PI x Brain Cell Annotations for covariance between the *g*-factor and psychotic disorders factor 52

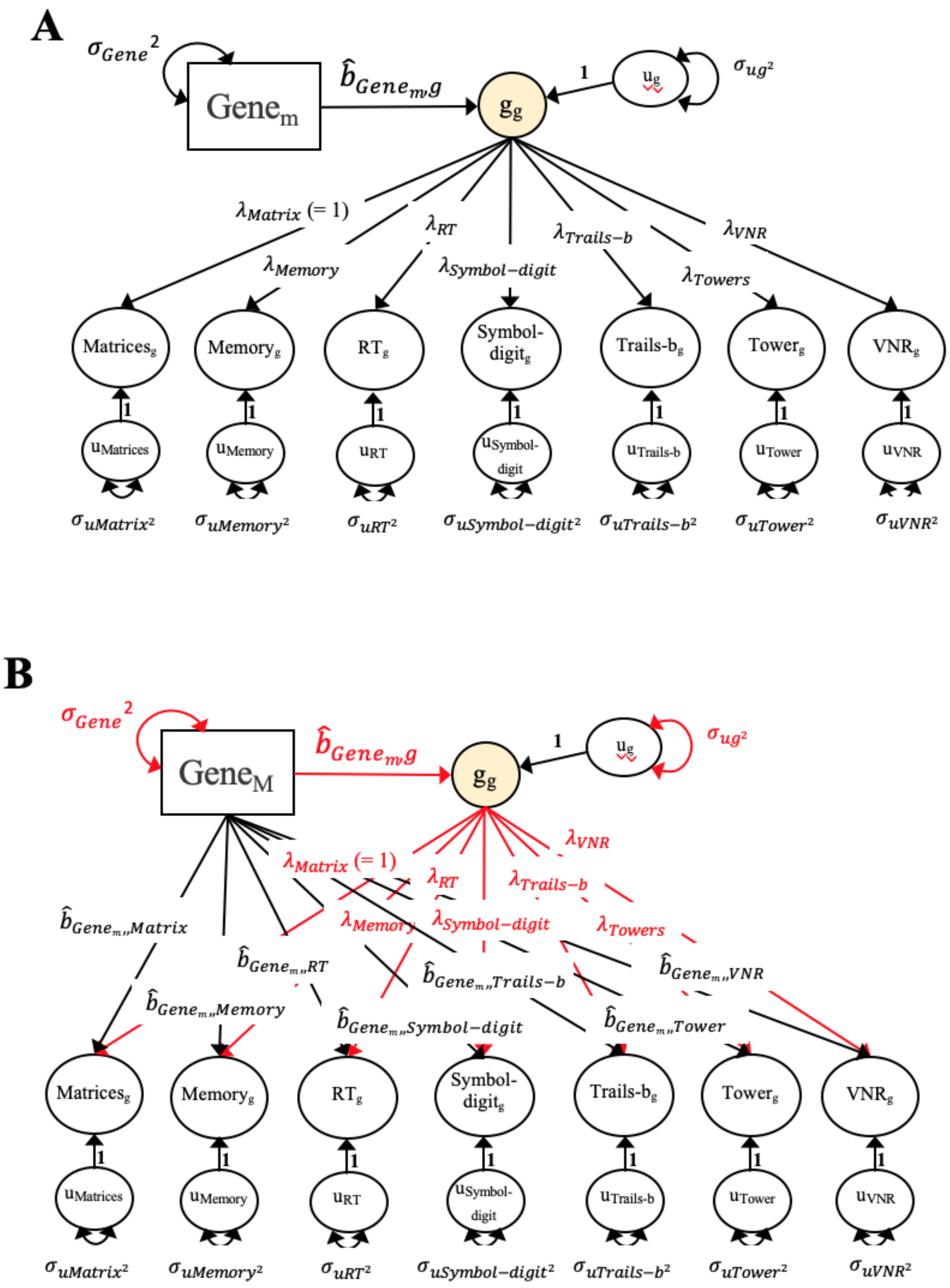

Supplementary Figure 1. T-SEM Models. *Panel A* depicts the model run to obtain estimates of each gene on the g-factor. This model is run *M* times to reflect the number of genes present across all traits, in this case 52,849 genes. *Panel B* depicts the model used to estimate Q_{Gene}, where Red lines and parameters are fixed from Step 1, and black lines and parameters are freely estimated in Step 2. The loading of the first indicator for each factor is fixed to 1 in all panels for identification purposes.

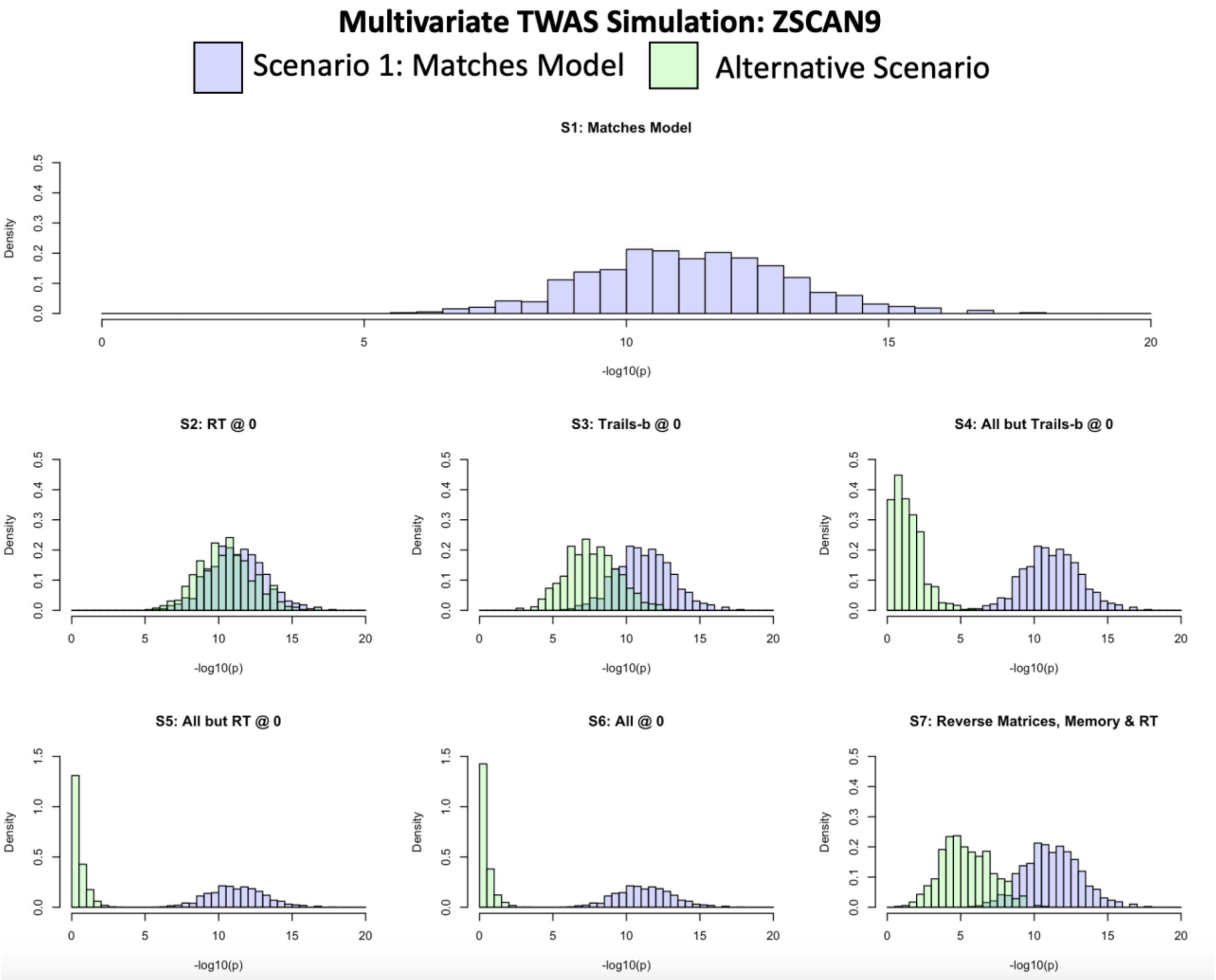

Supplementary Figure 2a. Histograms of T-SEM Simulation Results for ZSCAN9. Panels depict the two-sided -log10(p) values for estimated gene effects on the g-factor across the 7 different population generating scenarios when using the real data genetic covariance and sampling covariance matrix for ZSCAN9 in the cerebellum (the top hit for the *g*-factor) to sample simulated datasets (see **Online Method** for additional details). All panels depict in blue as a reference point the simulation scenario that exactly matched the factor model (i.e., Scenario 1 depicted in upper panel) and in green the specific scenario indicated in the histogram title.

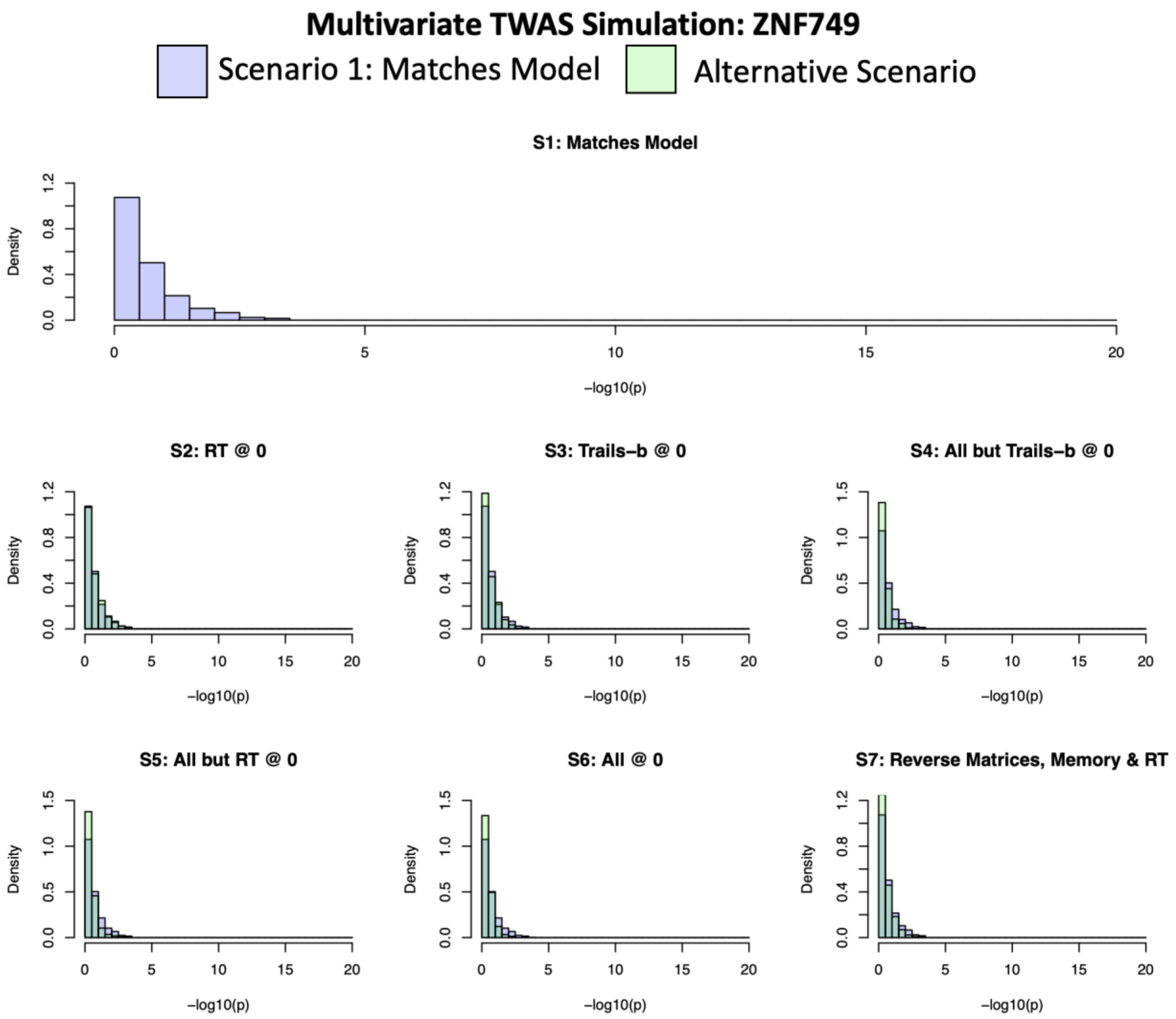

Supplementary Figure 2b. Histograms of T-SEM Simulation Results for ZNF749. Panels depict the two-sided -log10(p) values for estimated gene effects on the g-factor across the 7 different population generating scenarios when using the real data genetic covariance and sampling covariance matrix for ZNF749 in the hippocampus (the gene in the 50% percentile of *g*-factor results) to sample simulated datasets (see **Online Method** for additional details). All panels depict in blue as a reference point the simulation scenario that exactly matched the factor model (i.e., Scenario 1 depicted in upper panel) and in green the specific scenario indicated in the histogram title.

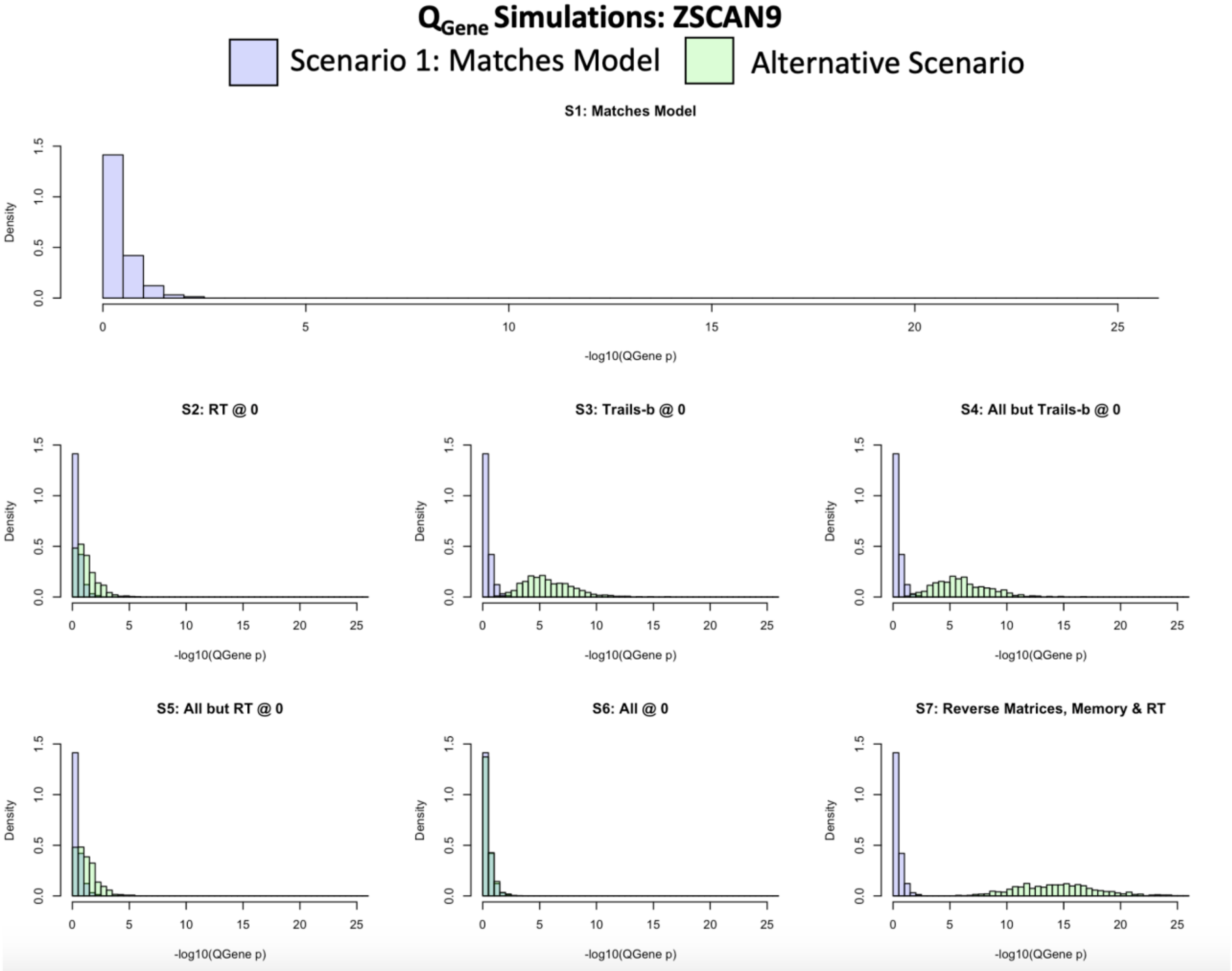

Supplementary Figure 3a. Histograms of Q_{Gene} Simulation Results for ZSCAN9. Panels depict the two-sided -log10(p) values for Q_{Gene} across the 7 different population generating scenario when using the real data genetic covariance and sampling covariance matrix for ZSCAN9 in the cerebellum (the top hit for the *g*-factor) to sample simulated datasets (see **Online Method** for additional details). All panels depict in blue as a reference point the simulation scenario that exactly matched the factor model (i.e., Scenario 1 depicted in upper panel) and in green the specific scenario indicated in the histogram title.

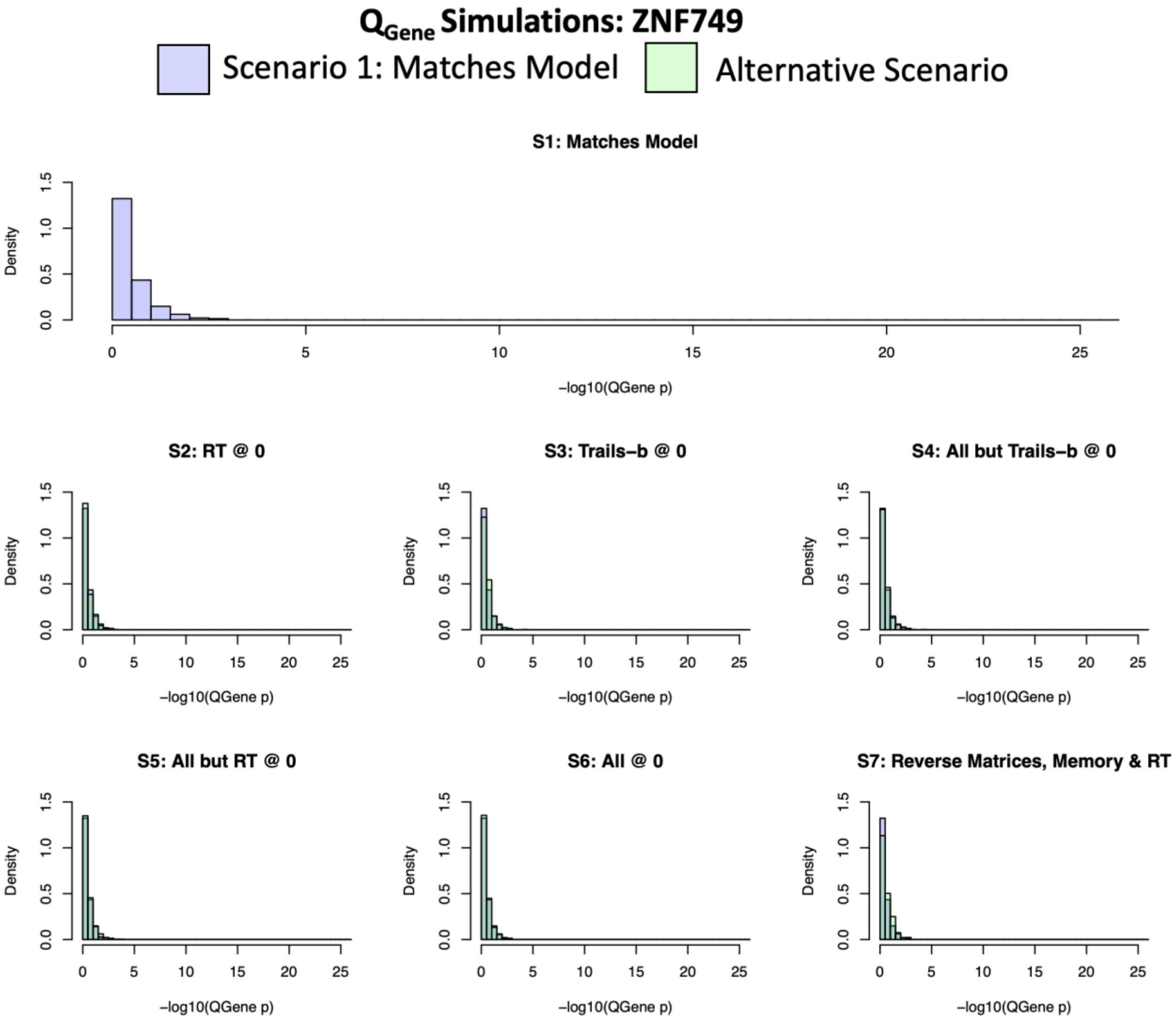

Supplementary Figure 3b. Histograms of Q_{Gene} Simulation Results for ZNF749. Panels depict the two-sided -log10(p) values for Q_{Gene} across the 7 different population generating scenarios when using the real data genetic covariance and sampling covariance matrix for ZNF749 in the hippocampus (the gene in the 50% percentile of *g*-factor results) to sample simulated datasets (see **Online Method** for additional details). All panels depict in blue as a reference point the simulation scenario that exactly matched the factor model (i.e., Scenario 1 depicted in upper panel) and in green the specific scenario indicated in the histogram title.

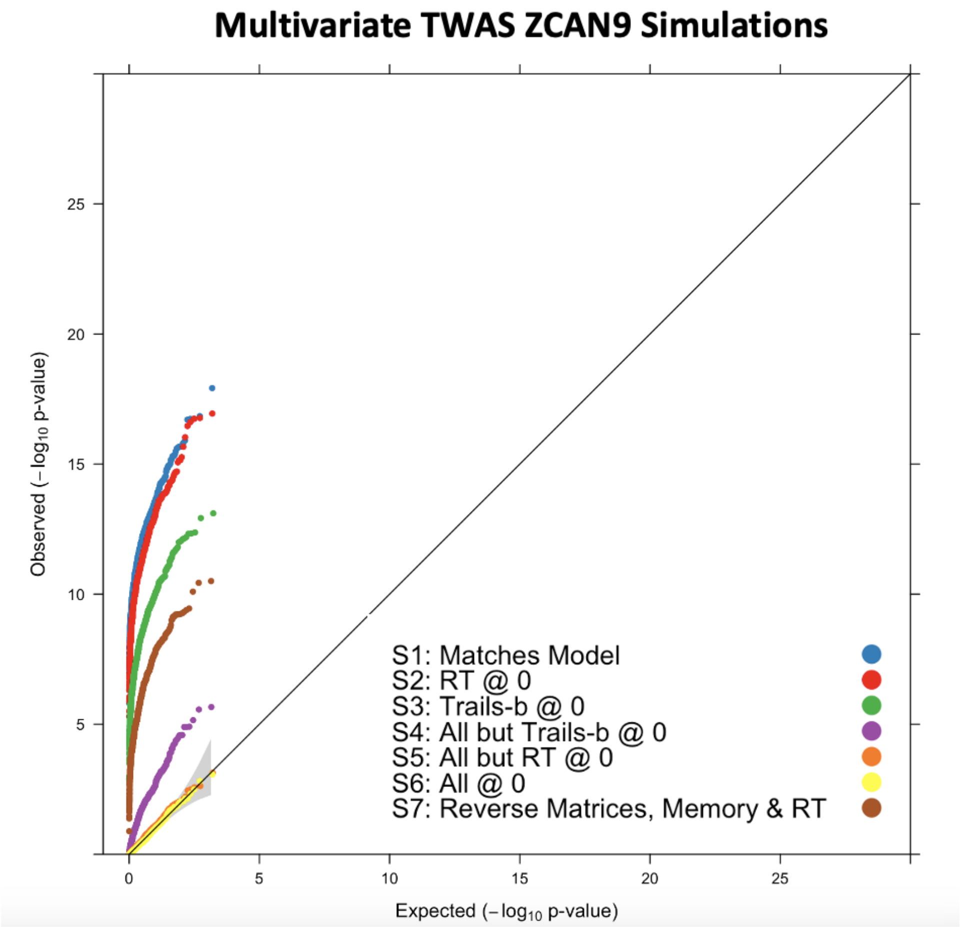

Supplementary Figure 4a. QQ-plot of T-SEM Simulation Results for ZCAN9. QQ-plot depicts the two-sided -log10(p) values for gene effects on the *g*-factor for the 7 different population generating scenarios when using the real data genetic covariance and sampling covariance matrix for ZSCAN9 in the cerebellum (the top hit for the *g*-factor) to sample simulated datasets (see **Online Method** for additional details). The 7 scenarios specifically reflect: Scenario 1 that matches the factor model depicted in blue; Scenario 2 with the covariance between the gene and reaction time (RT) set at 0 in the generating population in red; Scenario 3 with the covariance between the gene and Trails-b set at 0 in the generating population in green; Scenario 4 with the covariance between the gene and all traits except Trails-b set at 0 in the in the generating population in purple; Scenario 5 with the covariance between the SNP and all traits except RT at 0 in the generating population in orange; Scenario 6 with the covariance between the gene and all cognitive traits set at 0 in the generating population in yellow; and Scenario 7 with the covariance between the gene and Matrices, Memory, and RT traits directionally reversed in brown. Expected −log10(p)- values are those expected under the null hypothesis. The shaded area indicates the 95% confidence interval with the line on the diagonal indicating the null.

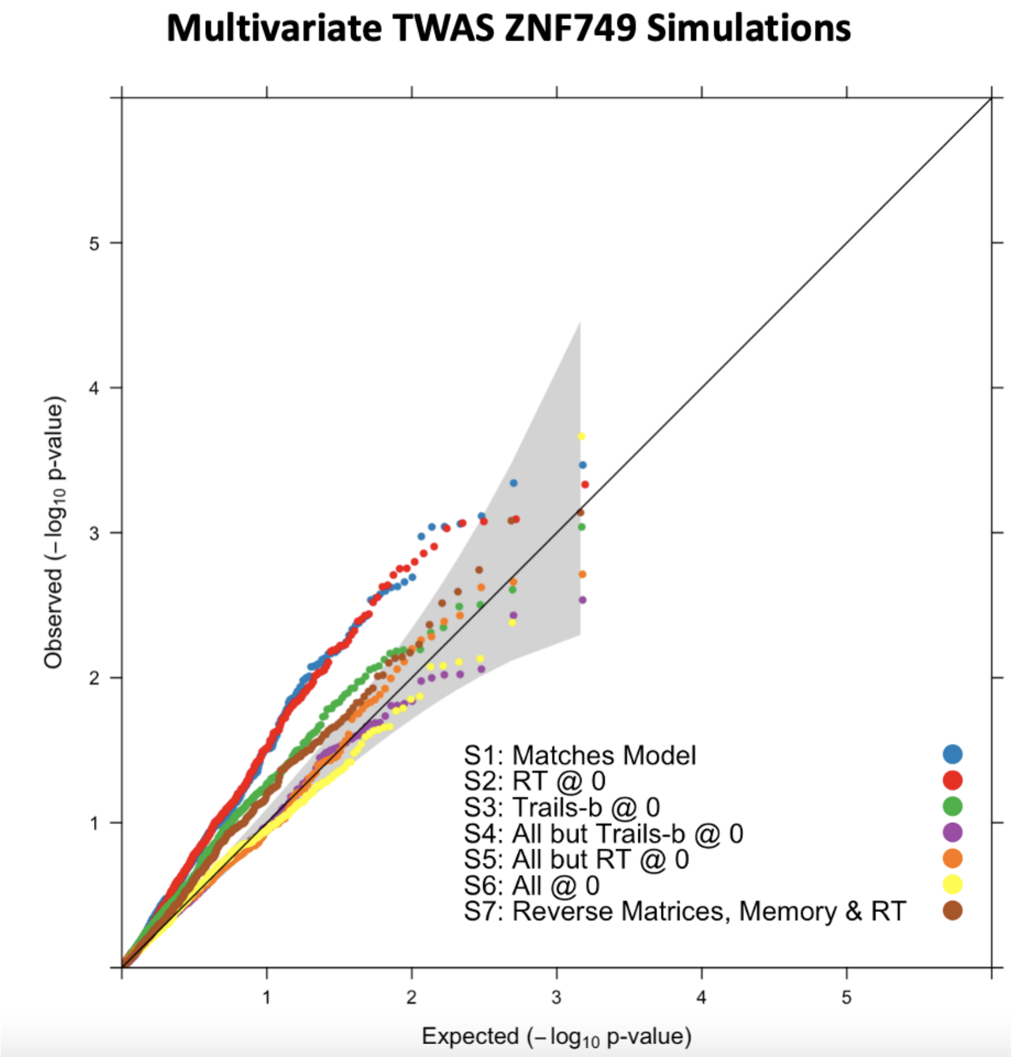

Supplementary Figure 4b. QQ-plot of T-SEM Simulation Results for ZNF749. QQ-plot depicts the two-sided -log10(p) values for gene effects on the g-factor for the 7 different population generating scenarios when using the real data genetic covariance and sampling covariance matrix for ZNF749 in the hippocampus (the gene in the 50% percentile of *g*-factor results) to sample simulated datasets (see **Online Method** for additional details). The 7 scenarios were specifically: Scenario 1 that matches the factor model depicted in blue; Scenario 2 with the covariance between the gene and reaction time (RT) set at 0 in the generating population in red; Scenario 3 with the covariance between the gene and Trails-b set at 0 in the generating population in green; Scenario 4 with the covariance between the gene and all traits except Trails-b set at 0 in the in the generating population in purple; Scenario 5 with the covariance between the SNP and all traits except RT at 0 in the generating population in orange; Scenario 6 with the covariance between the gene and all cognitive traits set at 0 in the generating population in yellow; and Scenario 7 with the covariance between the gene and Matrices, Memory, and RT traits directionally reversed in brown. Expected −log10(p)- values are those expected under the null hypothesis. The shaded area indicates the 95% confidence interval with the line on the diagonal indicating the null.

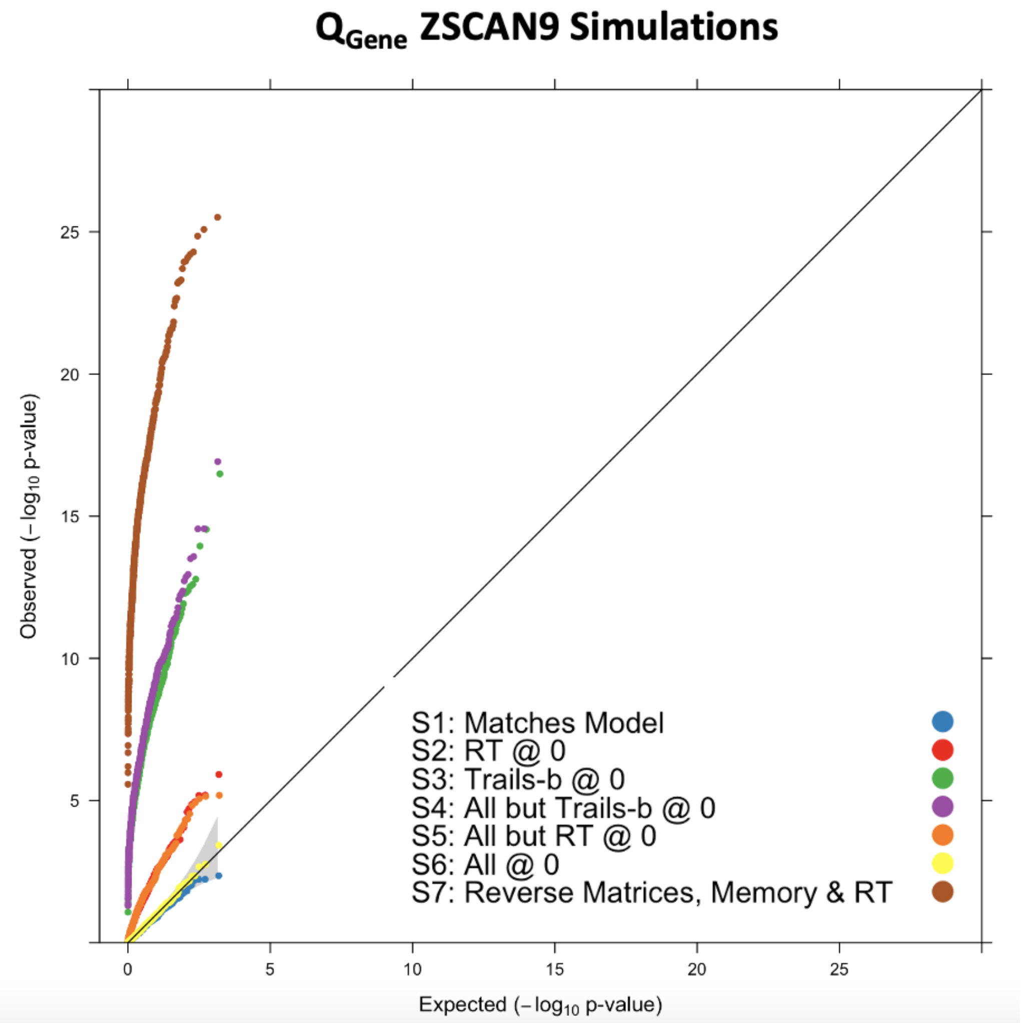

Supplementary Figure 5a. QQ-plot of Q_{Gene} Simulation Results for ZSCAN9. QQ-plot depicts the two-sided -log10(p) values for Q_{Gene} when using the real data genetic covariance and sampling covariance matrix for ZSCAN9 in the cerebellum (the top hit for the *g*-factor) to sample simulated datasets (see **Online Method** for additional details). The 7 scenarios specifically reflect: Scenario 1 that matches the factor model depicted in blue; Scenario 2 with the covariance between the gene and reaction time (RT) set at 0 in the generating population in red; Scenario 3 with the covariance between the gene and Trails-b set at 0 in the generating population in green; Scenario 4 with the covariance between the gene and all traits except Trails-b set at 0 in the in the generating population in purple; Scenario 5 with the covariance between the SNP and all traits except RT at 0 in the generating population in orange; Scenario 6 with the covariance between the gene and all cognitive traits set at 0 in the generating population in yellow; and Scenario 7 with the covariance between the gene and Matrices, Memory, and RT traits directionally reversed in brown. Expected −log10(p)- values are those expected under the null hypothesis. The shaded area indicates the 95% confidence interval with the line on the diagonal indicating the null.

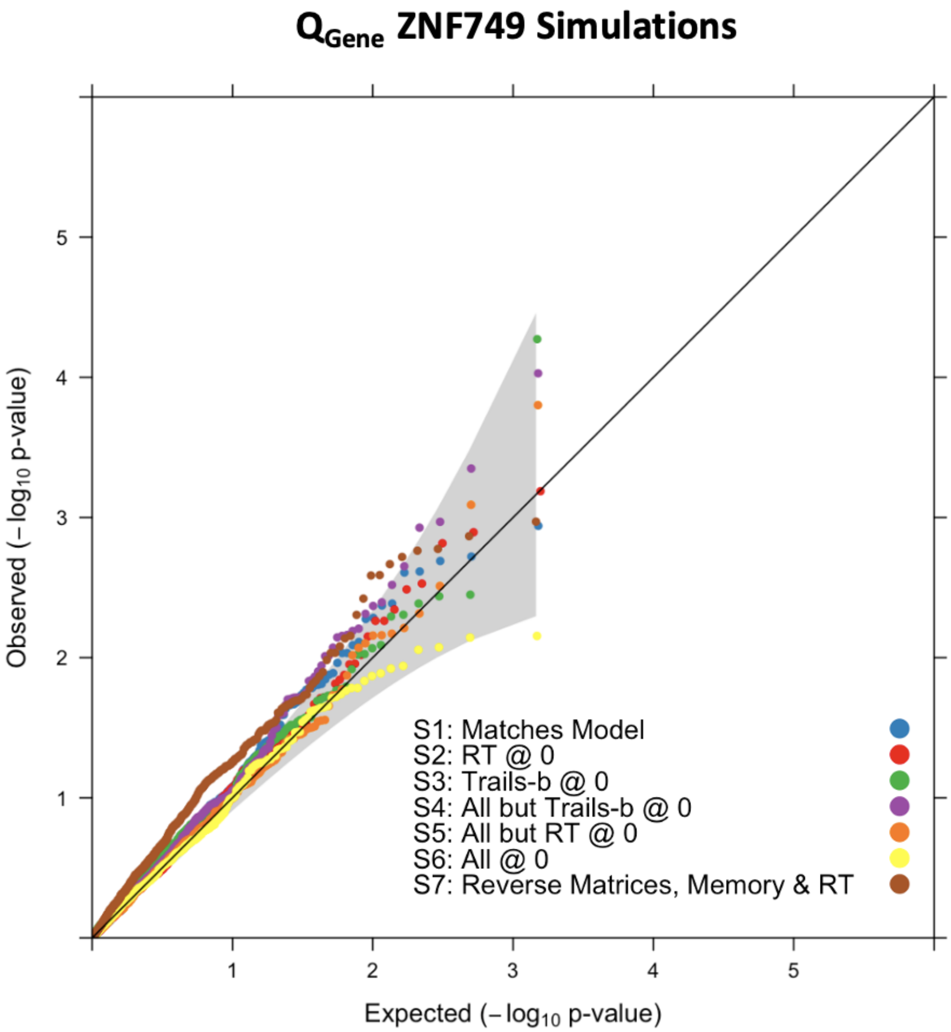

Supplementary Figure 5b. QQ-plot of Q_{Gene} Simulation Results for ZNF749. QQ-plot depicts the two-sided -log10(p) values for Q_{Gene} when using the real data genetic covariance and sampling covariance matrix for ZNF749 in the hippocampus (the gene in the 50% percentile of *g*-factor results) to sample simulated datasets (see **Online Method** for additional details). The 7 scenarios were specifically: Scenario 1 that matches the factor model depicted in blue; Scenario 2 with the covariance between the gene and reaction time (RT) set at 0 in the generating population in red; Scenario 3 with the covariance between the gene and Trails-b set at 0 in the generating population in green; Scenario 4 with the covariance between the gene and all traits except Trails-b set at 0 in the in the generating population in purple; Scenario 5 with the covariance between the SNP and all traits except RT at 0 in the generating population in orange; Scenario 6 with the covariance between the gene and all cognitive traits set at 0 in the generating population in yellow; and Scenario 7 with the covariance between the gene and Matrices, Memory, and RT traits directionally reversed in brown. Expected −log10(p)- values are those expected under the null hypothesis. The shaded area indicates the 95% confidence interval with the line on the diagonal indicating the null.

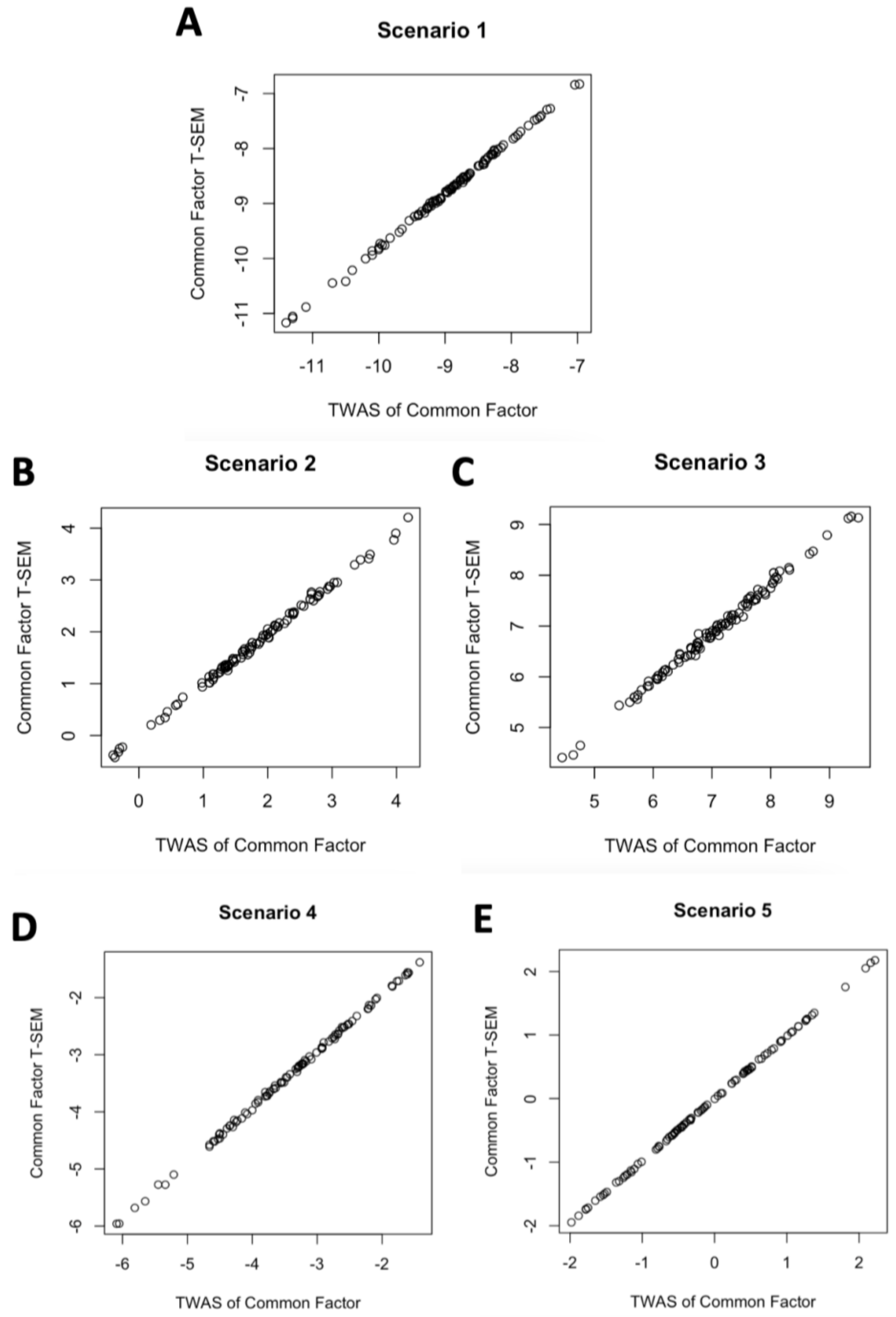

Supplementary Figure 6. Scatterplots of TWAS versus T-SEM Simulation Results. For all panels, the x-axis depicts Z-statistics for TWAS of the simulated g-factor summary statistics. The y-axis depicts Z-statistics for T-SEM of the common factor. Panel A depicts simulation results for Scenario 1 in which the population SNP effects matched the factor model. Panel B depicts simulations results for Scenario 2 in which the direction of the population SNP effects was reversed for three of the five indicators. Panel C depicts simulations results for Scenario 3 in which the population SNP effects was reversed and doubled for three of the five indicators. Panel D depicts simulation results for Scenario 4 in which the population SNP effects were set to 0 for three of the five indicators. Panel E depicts simulation results for Scenario 5 in which the population SNP effects were set to 0 for all five indicators.

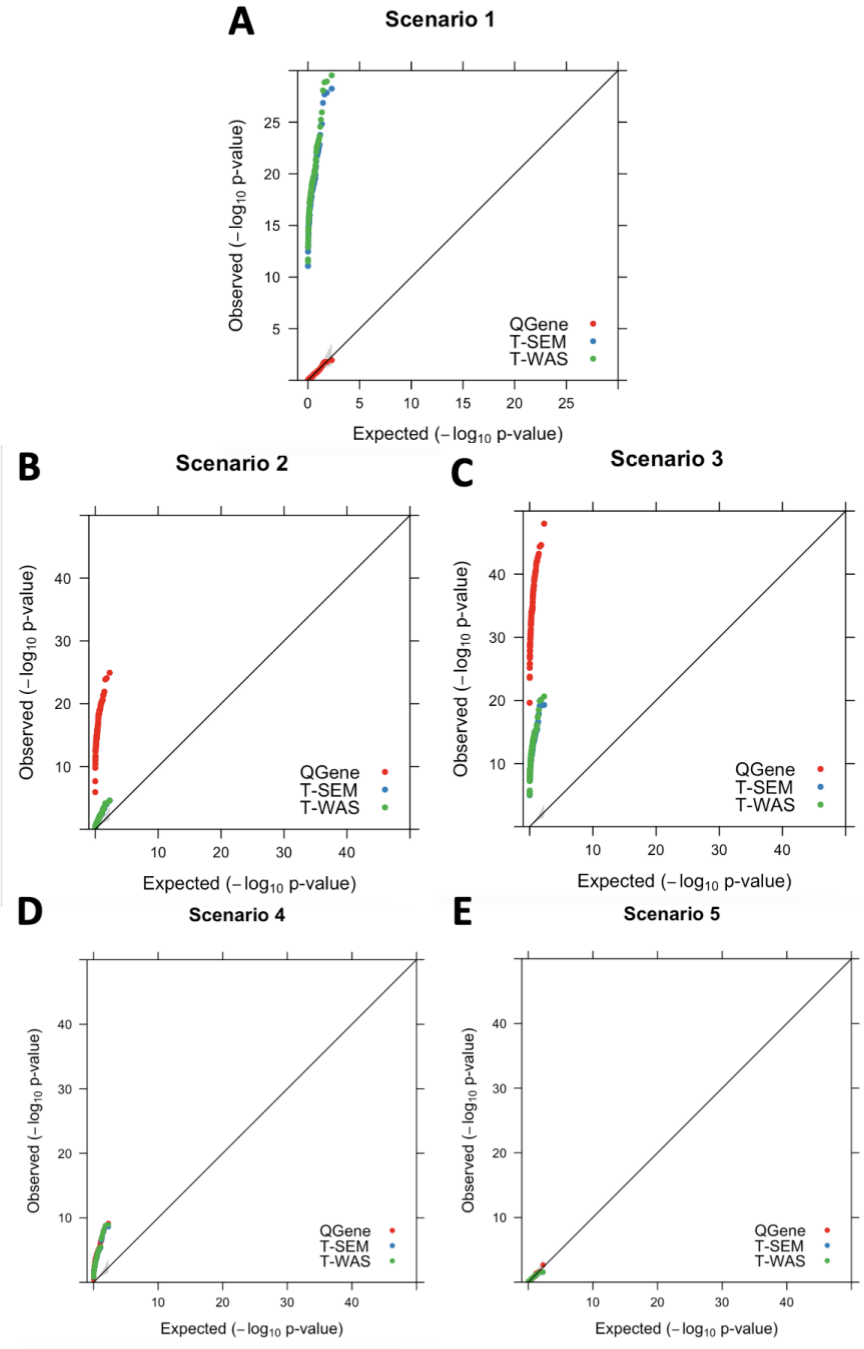

Supplementary Figure 7. QQ-plot of SNP-level Simulation Results. QQ-plots depict the two-sided -log10(p) values for TWAS of the common factor summary statistics (in green), T-SEM (in blue), and QGene (in red) for the five population generating scenarios. Note that for some panels only one set of dots is visible due to highly concordant findings across the sets of results. Panel A depicts simulation results for Scenario 1 in which the population SNP effects matched the factor model. Panel B depicts simulations results for Scenario 2 in which the direction of the population SNP effects was reversed for three of the five indicators. Panel C depicts simulations results for Scenario 3 in which the population SNP effects was reversed and doubled for three of the five indicators. Panel D depicts simulation results for Scenario 4 in which the population SNP effects were set to 0 for three of the five indicators. Panel E depicts simulation results for Scenario 5 in which the population SNP effects were set to 0 for all five indicators.

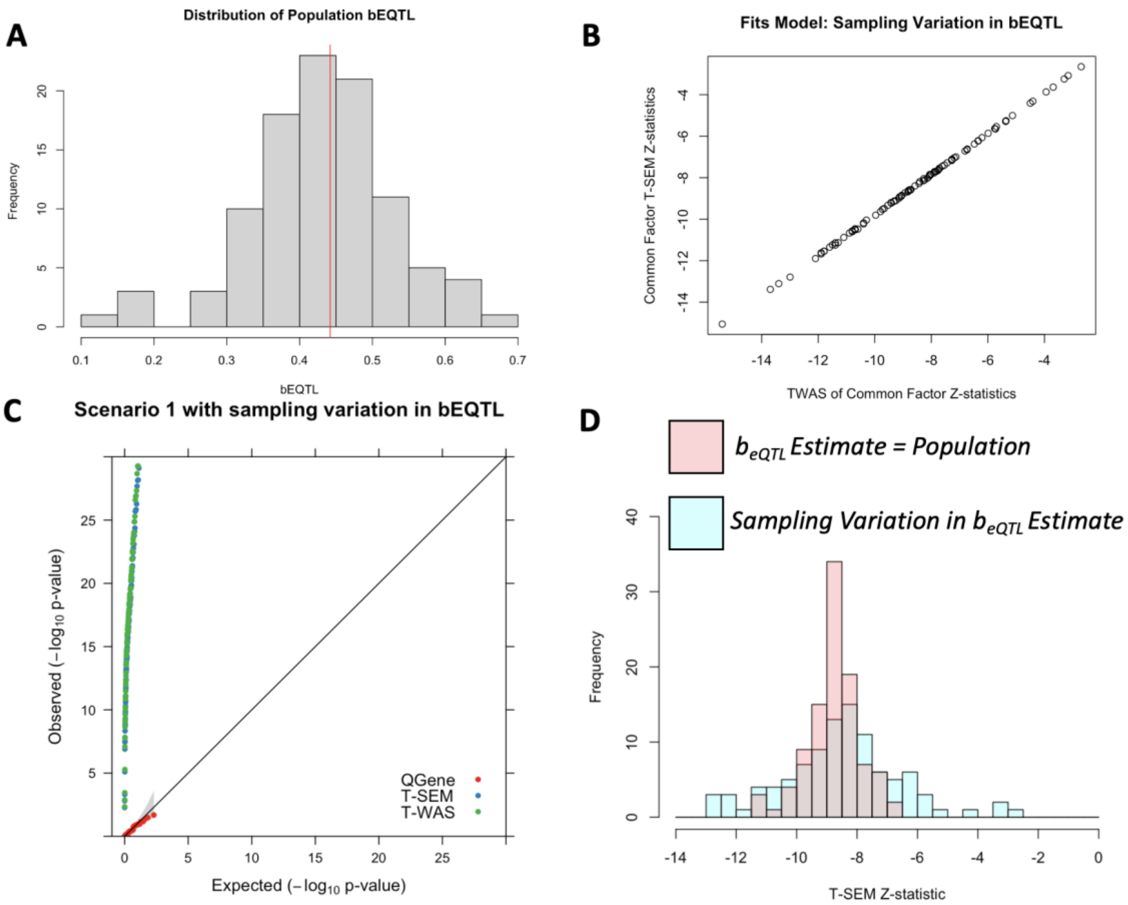

Supplementary Figure 8. Simulations Incorporating Sampling Variation in b_{eQTL} Estimates. ***Panel A*** depicts the distribution of population b_{eQTL} values across the 100 simulation runs. The red line reflects the b_{eQTL} estimated from a finite sample used by FUSION, with values farther from the line indicating greater discordance across the FUSION weights and population values. ***Panel B*** depicts the concordance across the TWAS and T-SEM *Z*-statistics; in line with the other simulations, these results are highly concordant. ***Panel C*** depicts the QQ-plot of the two-sided -log10(p-values) for TWAS results in green, the T-SEM results in blue, and the Q_{Gene} results in red. T-SEM and TWAS are both well-powered, as would be expected given a generating population that matched the factor model. Also in line with expectation, Q_{Gene} does not deviate from the null. ***Panel D*** displays the overlaid histograms of estimates obtained without including sampling variation for b_{eQTL} estimates in red along with estimates obtained with sampling variation in blue. As would be expected, including this aspect of sampling variation serves to generally increase variability in T-SEM *Z*-statistics.

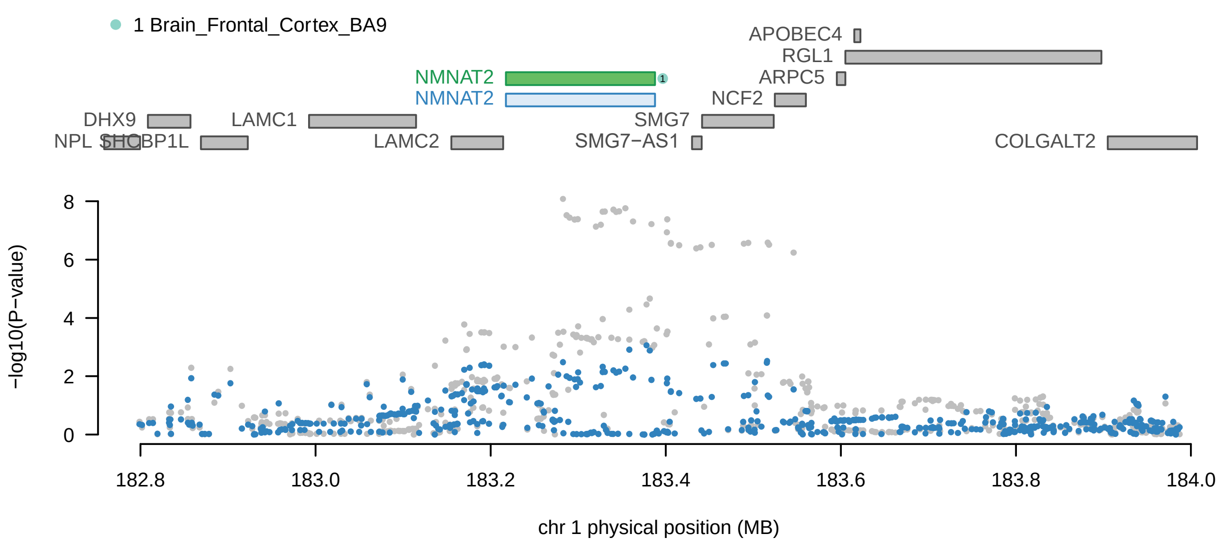

Supplementary Figure 9a. Regional Association Plot for Locus 1 from g-factor Conditional Analyses. The top half of the panel displays all of the genes located in the locus window. Genes that were marginally significant are highlighted in blue while those that were jointly significant are highlighted in green. The color and numbering of the point next to jointly significant green genes indicates the specific tissue type (legend in upper-left) for which that gene was jointly significant. The bottom half depicts the Manhattan plot of the GWAS SNP effects within the locus prior to (grey) and after (blue) conditioning on the jointly significant genes in green. There were no genome-wide significant effects within this locus after conditioning on the predicted expression of NMNAT2 in the frontal cortex. Dots are vertically positioned along the y-axis of the Manhattan plot according to their two-sided -log10(p-values).

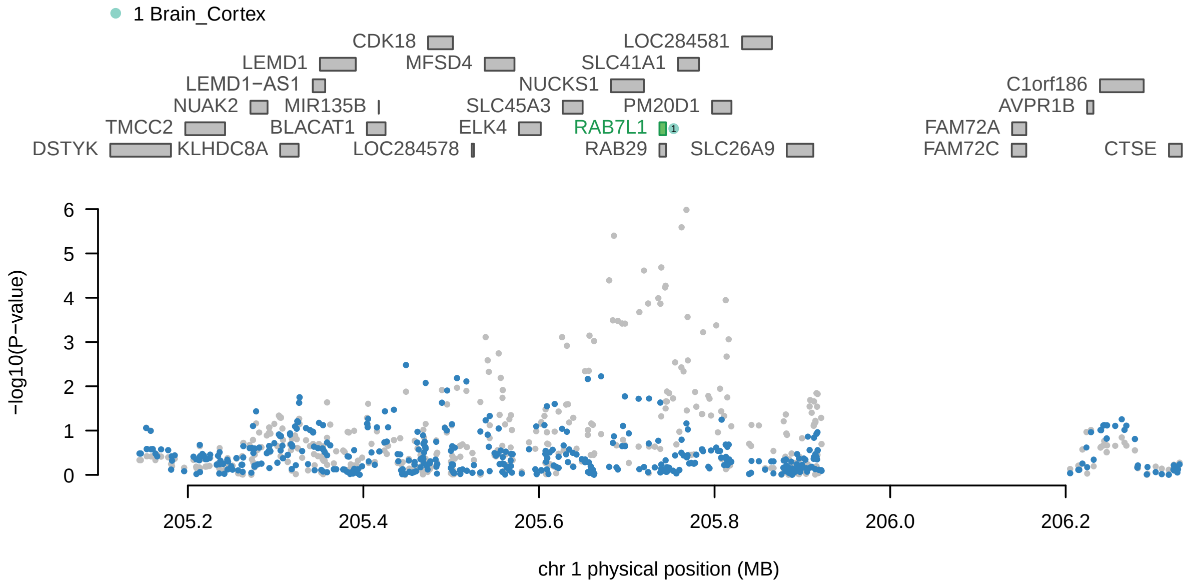

Supplementary Figure 9b. Regional Association Plot for Locus 2 from g-factor Conditional Analyses. The top half of the panel displays all of the genes located in the locus window. Genes that were marginally significant are highlighted in blue while those that were jointly significant are highlighted in green. The color and numbering of the point next to jointly significant green genes indicates the specific tissue type (legend in upper-left) for which that gene was jointly significant. The bottom half depicts the Manhattan plot of the GWAS SNP effects within the locus prior to (grey) and after (blue) conditioning on the jointly significant genes in green. There were no marginally significant genome-wide effects within this locus after conditioning on the predicted expression of RAB7L1 in the cortex. Dots are vertically positioned along the y-axis of the Manhattan plot according to their two-sided -log10(p-values).

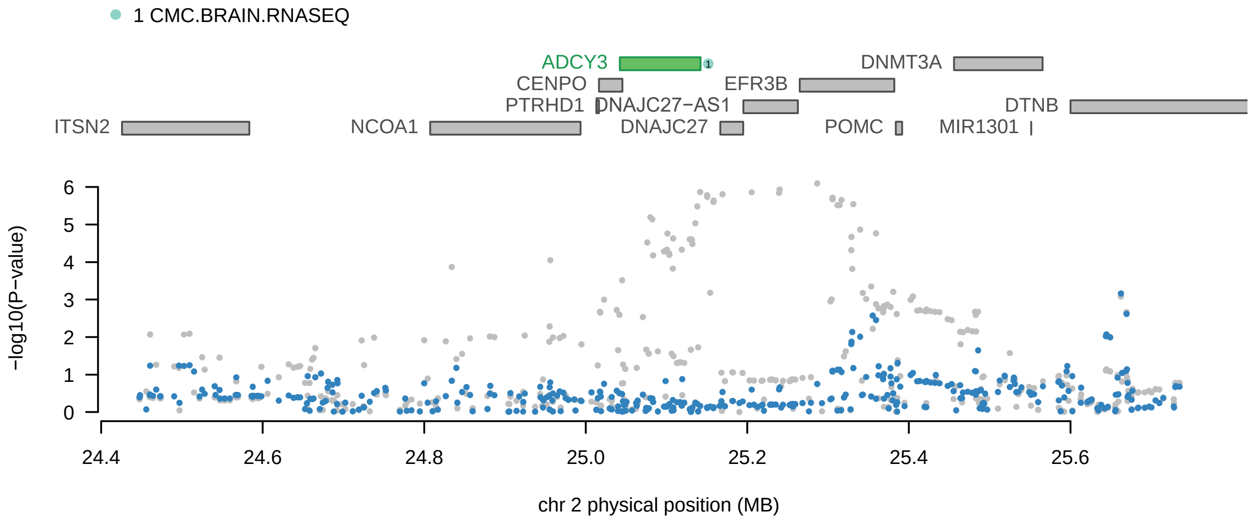

Supplementary Figure 9c. Regional Association Plot for Locus 3 from g-factor Conditional Analyses. The top half of the panel displays all of the genes located in the locus window. Genes that were marginally significant are highlighted in blue while those that were jointly significant are highlighted in green. The color and numbering of the point next to jointly significant green genes indicates the specific tissue type (legend in upper-left) for which that gene was jointly significant. The bottom half depicts the Manhattan plot of the GWAS SNP effects within the locus prior to (grey) and after (blue) conditioning on the jointly significant genes in green. There were no marginally significant genome-wide effects within this locus after conditioning on the predicted expression of ADCY3 in the Common Mind Consortium RNA-seq dlPFC tissue type. Dots are vertically positioned along the y-axis of the Manhattan plot according to their two-sided -log10(p-values).

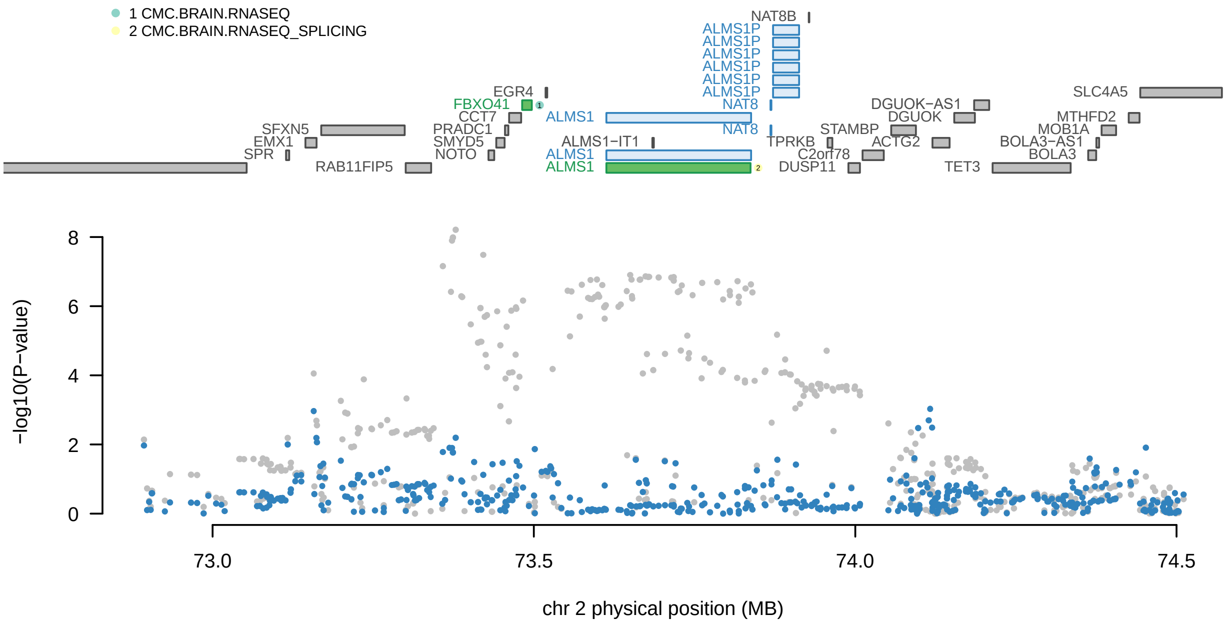

Supplementary Figure 9d. Regional Association Plot for Locus 4 from g-factor Conditional Analyses. The top half of the panel displays all of the genes located in the locus window. Genes that were marginally significant are highlighted in blue while those that were jointly significant are highlighted in green. The color and numbering of the point next to jointly significant green genes indicates the specific tissue type (legend in upper-left) for which that gene was jointly significant. The bottom half depicts the Manhattan plot of the GWAS SNP effects within the locus prior to (grey) and after (blue) conditioning on the jointly significant genes in green. There were no significant genome-wide effects within this locus after conditioning on the predicted expression of FBXO41 gene in the Common Mind Consortium RNA-seq dlPFC tissue type and the ALMS1 gene in the Common Mind Consortium RNA-seq splicing dlPFC tissue type. Dots are vertically positioned along the y-axis of the Manhattan plot according to their two-sided -log10(p-values).

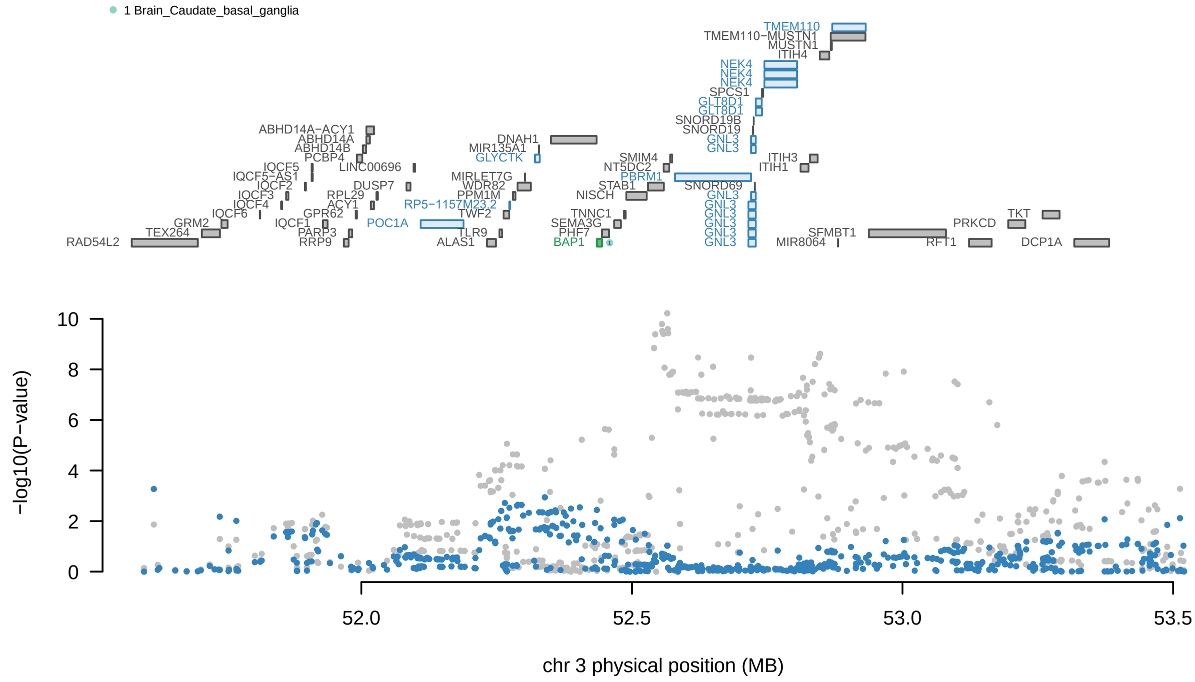

Supplementary Figure 9e. Regional Association Plot for Locus 6 from g-factor Conditional Analyses. The top half of the panel displays all of the genes located in the locus window. Genes that were marginally significant are highlighted in blue while those that were jointly significant are highlighted in green. The color and numbering of the point next to jointly significant green genes indicates the specific tissue type (legend in upper-left) for which that gene was jointly significant. The bottom half depicts the Manhattan plot of the GWAS SNP effects within the locus prior to (grey) and after (blue) conditioning on the jointly significant genes in green. There were no significant genome-wide effects within this locus after conditioning on the predicted expression of BAP1 gene in the caudate tissue type. Dots are vertically positioned along the y-axis of the Manhattan plot according to their two-sided -log10(p-values).

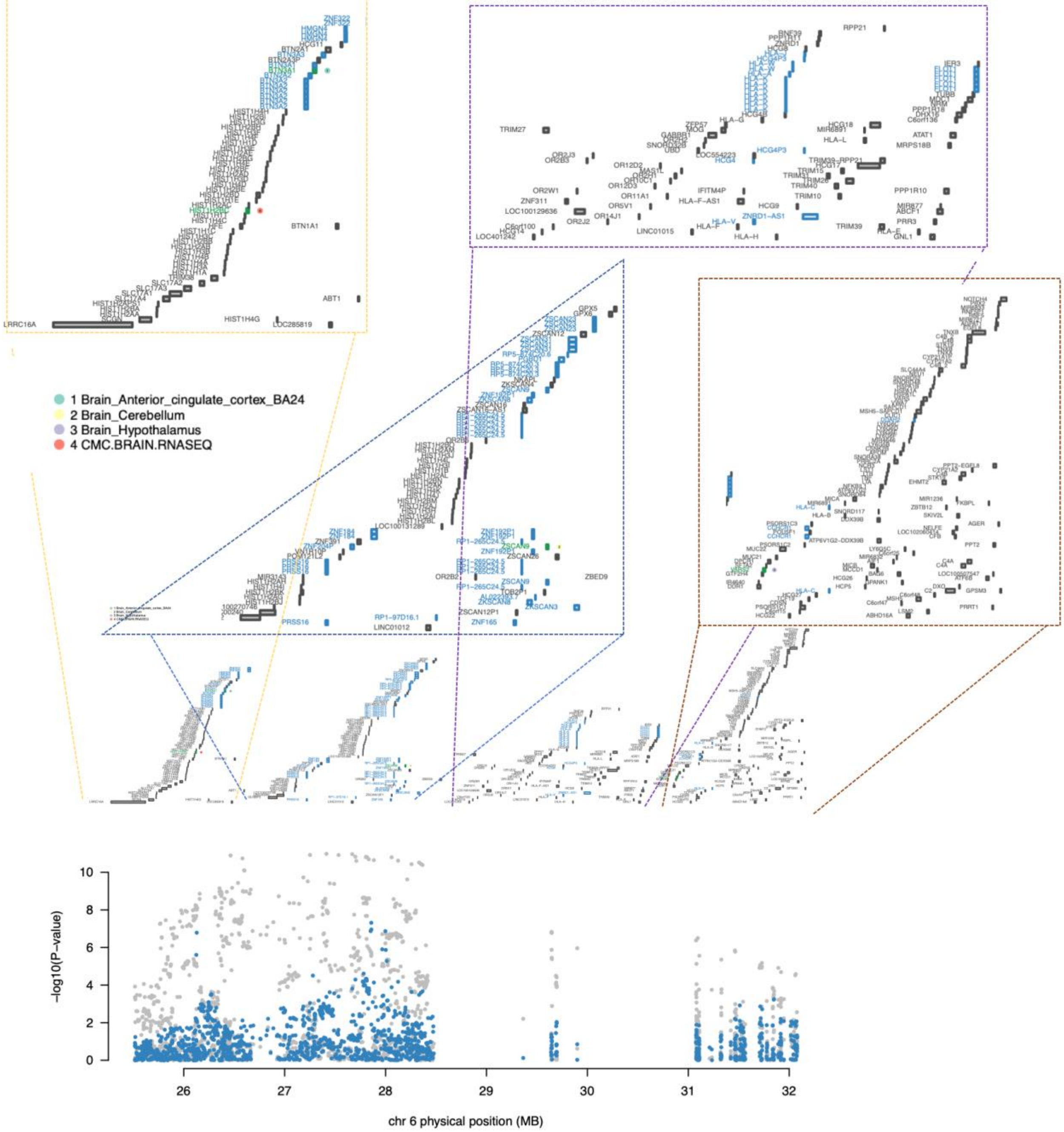

Supplementary Figure 9f. Regional Association Plot for Locus 7 from g-factor Conditional Analyses. The top half of the panel displays all of the genes located in the locus window. Genes that were marginally significant are highlighted in blue while those that were jointly significant are highlighted in green. The color and numbering of the point next to jointly significant green genes indicates the specific tissue type (legend in upper-left) for which that gene was jointly significant. The bottom half depicts the Manhattan plot of the GWAS SNP effects within the locus prior to (grey) and after (blue) conditioning on the jointly significant genes in green. There were no significant genome-wide effects within this locus after conditioning on the predicted expression of HIST1H2Bc gene in the Common Mind Consortium RNA-seq dlPFC tissue type tissue type, the ZSCAN9 gene in the cerebellum, the VARS2 gene in the hypothalamus and the BTN3A1 gene in the anterior cingulate cortex. Due to the number of genes within this particular locus, zoomed in portions of the top half of the plot are provided directly above. Dots are vertically positioned along the y-axis of the Manhattan plot according to their two-sided -log10(p-values).

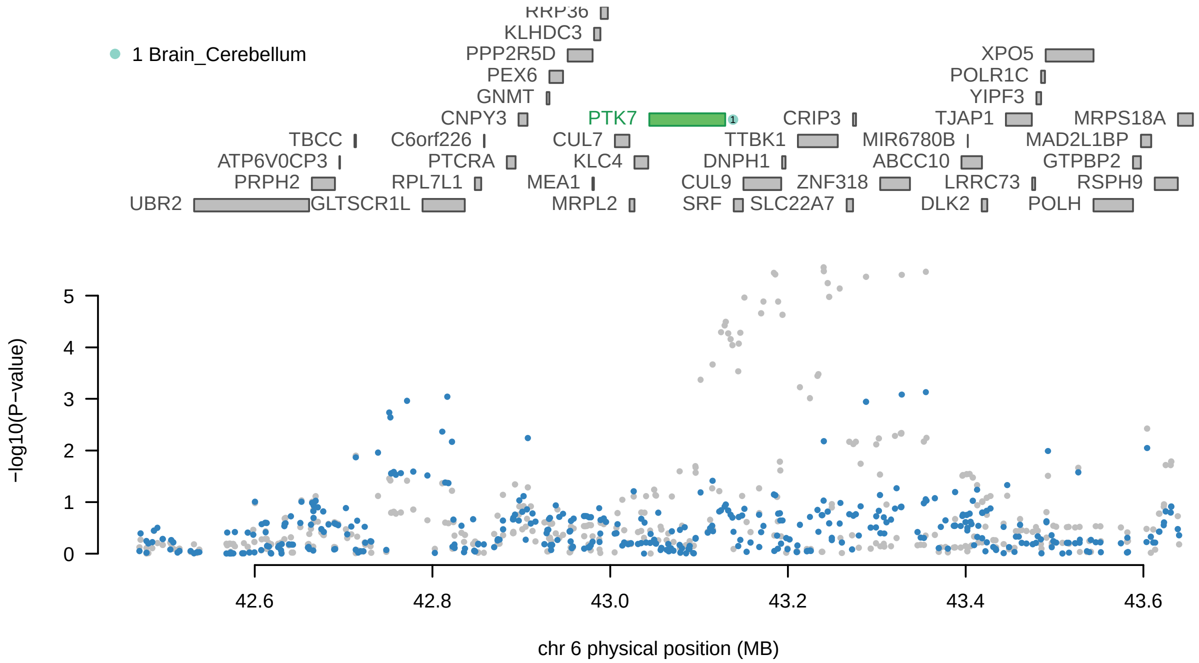

Supplementary Figure 9g. Regional Association Plot for Locus 8 from g-factor Conditional Analyses. The top half of the panel displays all of the genes located in the locus window. Genes that were marginally significant are highlighted in blue while those that were jointly significant are highlighted in green. The color and numbering of the point next to jointly significant green genes indicates the specific tissue type (legend in upper-left) for which that gene was jointly significant. The bottom half depicts the Manhattan plot of the GWAS SNP effects within the locus prior to (grey) and after (blue) conditioning on the jointly significant genes in green. There were no marginally significant genome-wide effects within this locus after conditioning on the predicted expression of PTK7 gene in the cerebellum tissue type. Dots are vertically positioned along the y-axis of the Manhattan plot according to their two-sided -log10(p-values).

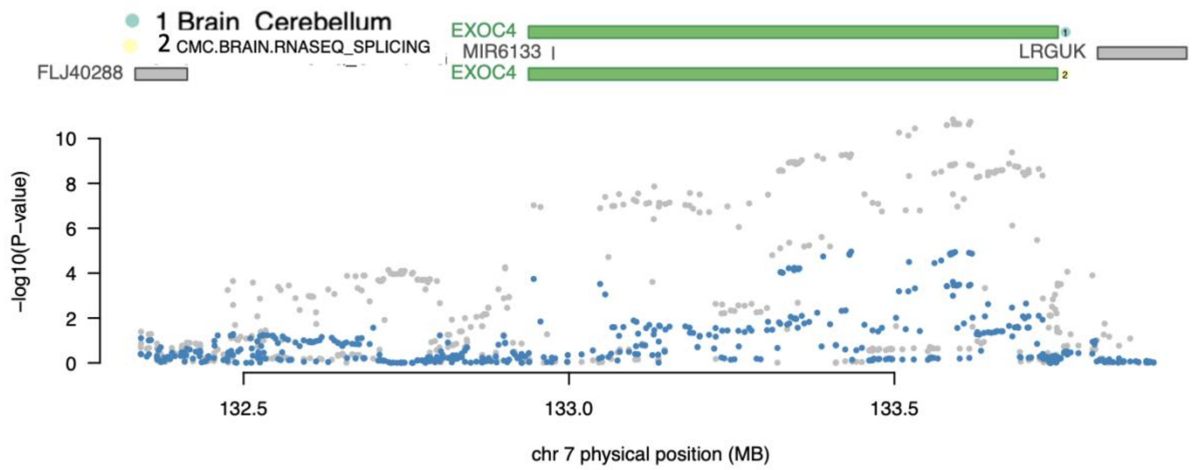

Supplementary Figure 9h. Regional Association Plot for Locus 9 from g-factor Conditional Analyses. The top half of the panel displays all of the genes located in the locus window. Genes that were marginally significant are highlighted in blue while those that were jointly significant are highlighted in green. The color and numbering of the point next to jointly significant green genes indicates the specific tissue type (legend in upper-left) for which that gene was jointly significant. The bottom half depicts the Manhattan plot of the GWAS SNP effects within the locus prior to (grey) and after (blue) conditioning on the jointly significant genes in green. There were no marginally significant genome-wide effects within this locus after conditioning on the predicted expression of EXOC4 gene in both the cerebellum and Common Mind Consortium RNA-seq dlPFC tissue types. Dots are vertically positioned along the y-axis of the Manhattan plot according to their two-sided -log10(p-values).

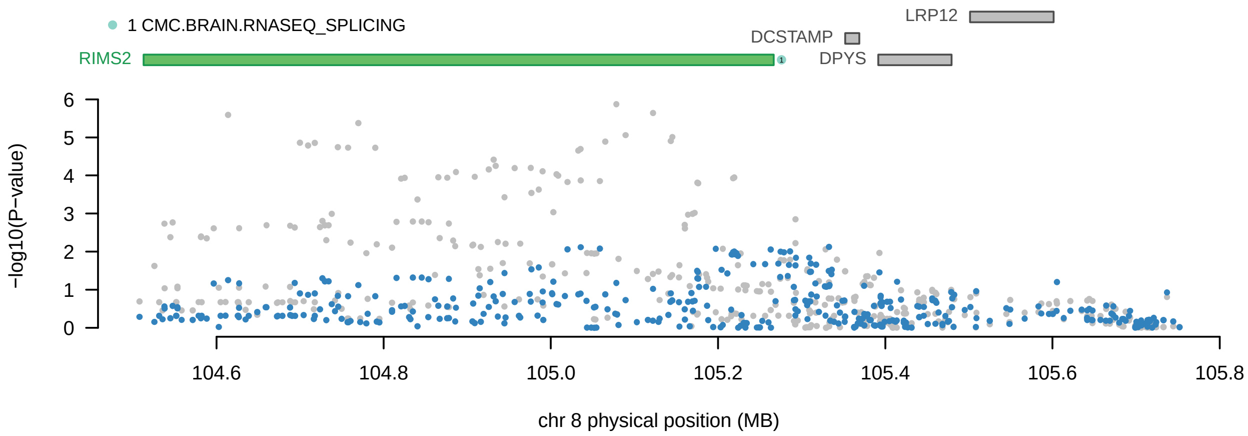

Supplementary Figure 9i. Regional Association Plot for Locus 10 from g-factor Conditional Analyses. The top half of the panel displays all of the genes located in the locus window. Genes that were marginally significant are highlighted in blue while those that were jointly significant are highlighted in green. The color and numbering of the point next to jointly significant green genes indicates the specific tissue type (legend in upper-left) for which that gene was jointly significant. The bottom half depicts the Manhattan plot of the GWAS SNP effects within the locus prior to (grey) and after (blue) conditioning on the jointly significant genes in green. There were no marginally significant genome-wide effects within this locus after conditioning on the predicted expression of RIMS2 gene in the Common Mind Consortium RNA-seq dlPFC tissue type. Dots are vertically positioned along the y-axis of the Manhattan plot according to their two-sided -log10(p-values).

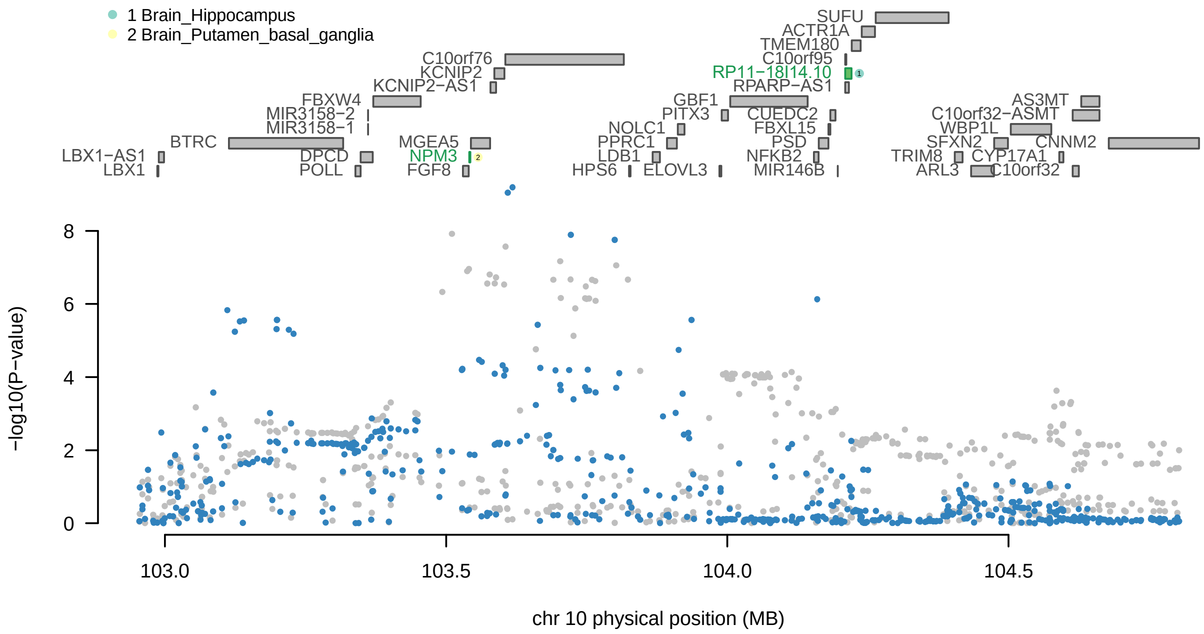

Supplementary Figure 9j. Regional Association Plot for Locus 11 from g-factor Conditional Analyses. The top half of the panel displays all of the genes located in the locus window. Genes that were marginally significant are highlighted in blue while those that were jointly significant are highlighted in green. The color and numbering of the point next to jointly significant green genes indicates the specific tissue type (legend in upper-left) for which that gene was jointly significant. The bottom half depicts the Manhattan plot of the GWAS SNP effects within the locus prior to (grey) and after (blue) conditioning on the jointly significant genes in green. This particular locus reflects a unique case where some conditioned GWAS effects are more significant than unconditioned effects. This can occur when there is mismatch within a region between the LD reference and GWAS data. Dots are vertically positioned along the y-axis of the Manhattan plot according to their two-sided -log10(p-values).

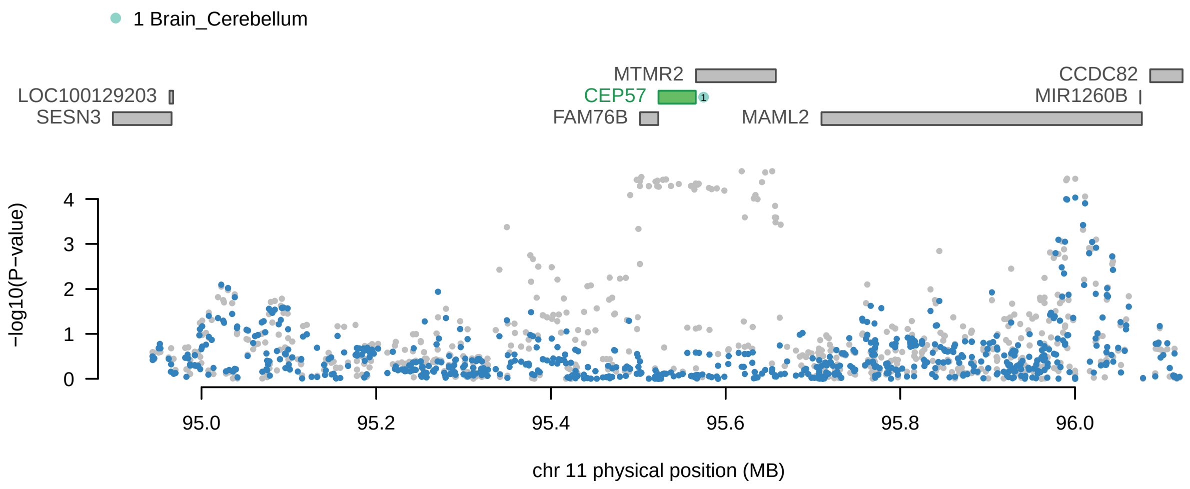

Supplementary Figure 9k. Regional Association Plot for Locus 12 from g-factor Conditional Analyses. The top half of the panel displays all of the genes located in the locus window. Genes that were marginally significant are highlighted in blue while those that were jointly significant are highlighted in green. The color and numbering of the point next to jointly significant green genes indicates the specific tissue type (legend in upper-left) for which that gene was jointly significant. The bottom half depicts the Manhattan plot of the GWAS SNP effects within the locus prior to (grey) and after (blue) conditioning on the jointly significant genes in green. There were no marginally significant genome-wide effects within this locus after conditioning on the predicted expression of CEP57 gene in the cerebellum. Dots are vertically positioned along the y-axis of the Manhattan plot according to their two-sided -log10(p-values).

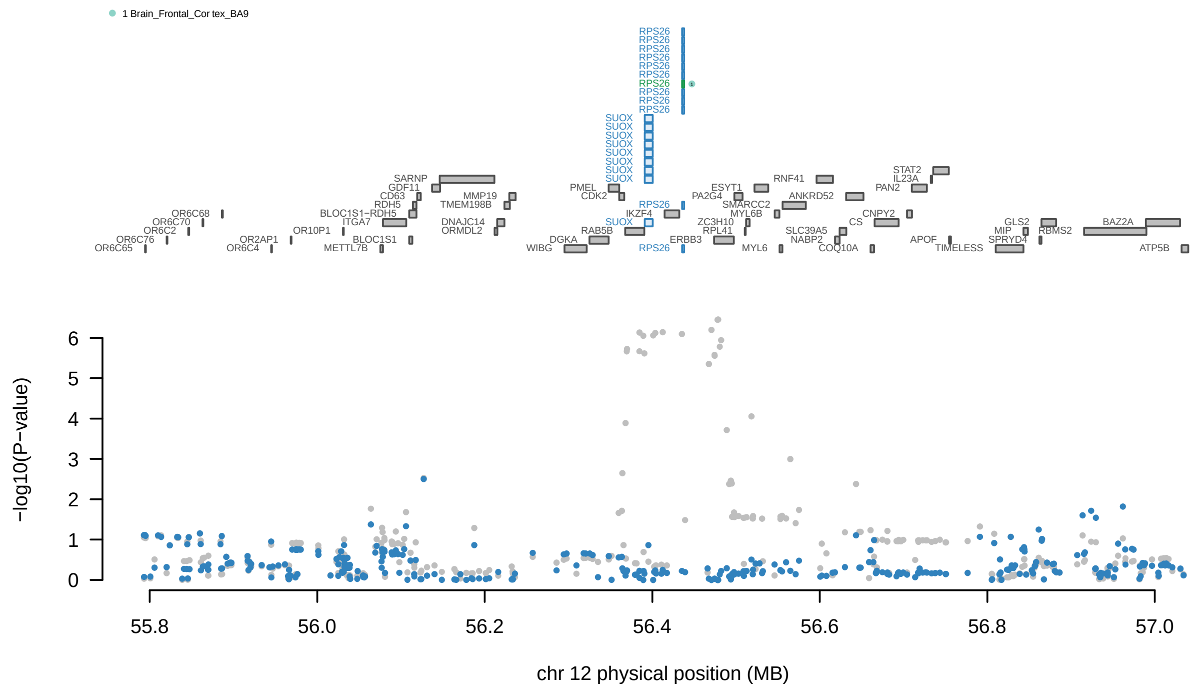

Supplementary Figure 9l. Regional Association Plot for Locus 13 from g-factor Conditional Analyses. The top half of the panel displays all of the genes located in the locus window. Genes that were marginally significant are highlighted in blue while those that were jointly significant are highlighted in green. The color and numbering of the point next to jointly significant green genes indicates the specific tissue type (legend in upper-left) for which that gene was jointly significant. The bottom half depicts the Manhattan plot of the GWAS SNP effects within the locus prior to (grey) and after (blue) conditioning on the jointly significant genes in green. There were no marginally significant genome-wide effects within this locus after conditioning on the predicted expression of the RPS26 gene in the frontal cortex. Dots are vertically positioned along the y-axis of the Manhattan plot according to their two-sided -log10(p-values).

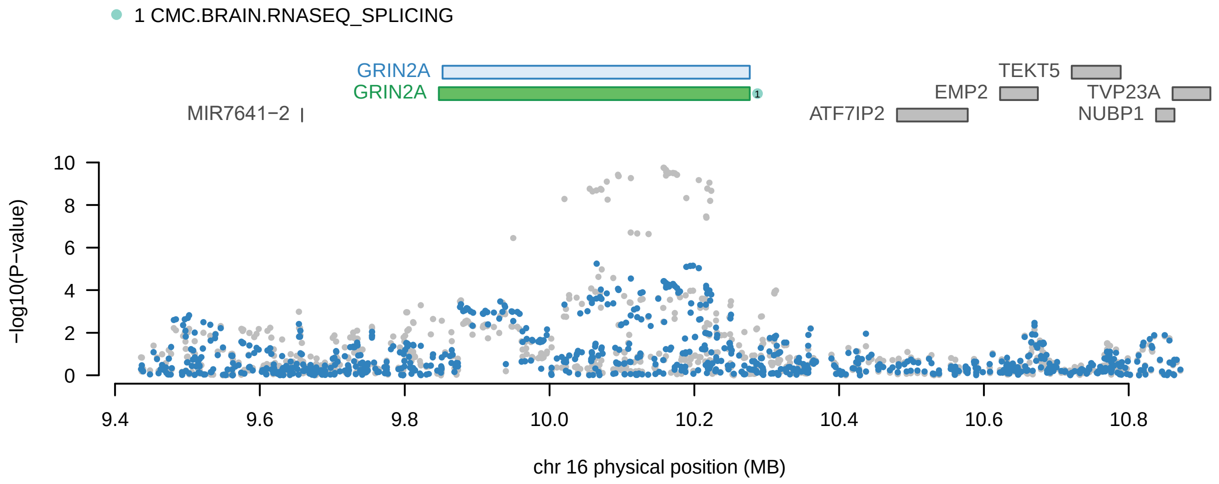

Supplementary Figure 9m. Regional Association Plot for Locus 14 from g-factor Conditional Analyses. The top half of the panel displays all of the genes located in the locus window. Genes that were marginally significant are highlighted in blue while those that were jointly significant are highlighted in green. The color and numbering of the point next to jointly significant green genes indicates the specific tissue type (legend in upper-left) for which that gene was jointly significant. The bottom half depicts the Manhattan plot of the GWAS SNP effects within the locus prior to (grey) and after (blue) conditioning on the jointly significant genes in green. There were no significant genome-wide effects within this locus after conditioning on the predicted expression of the GRIN2A gene in the Common Mind Consortium RNA-seq dlPFC tissue. Dots are vertically positioned along the y-axis of the Manhattan plot according to their two-sided -log10(p-values).

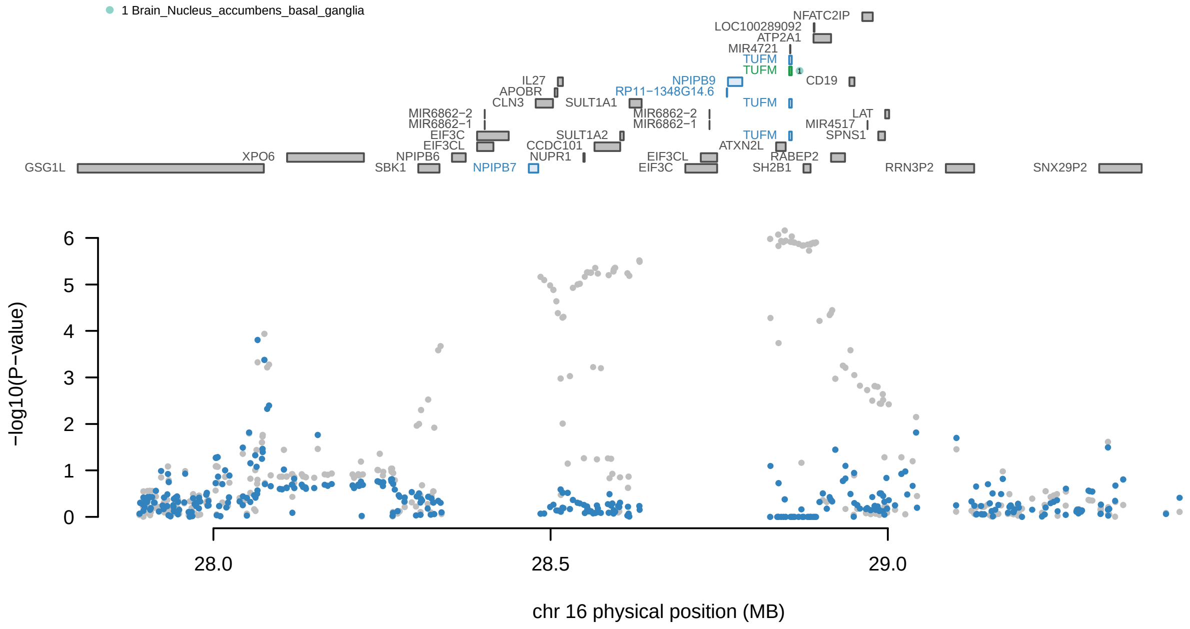

Supplementary Figure 9n. Regional Association Plot for Locus 15 from g-factor Conditional Analyses. The top half of the panel displays all of the genes located in the locus window. Genes that were marginally significant are highlighted in blue while those that were jointly significant are highlighted in green. The color and numbering of the point next to jointly significant green genes indicates the specific tissue type (legend in upper-left) for which that gene was jointly significant. The bottom half depicts the Manhattan plot of the GWAS SNP effects within the locus prior to (grey) and after (blue) conditioning on the jointly significant genes in green. There were no marginally significant genome-wide effects within this locus after conditioning on the predicted expression of the TUFM gene in the nucleus accumbens. Dots are vertically positioned along the y-axis of the Manhattan plot according to their two-sided -log10(p-values).

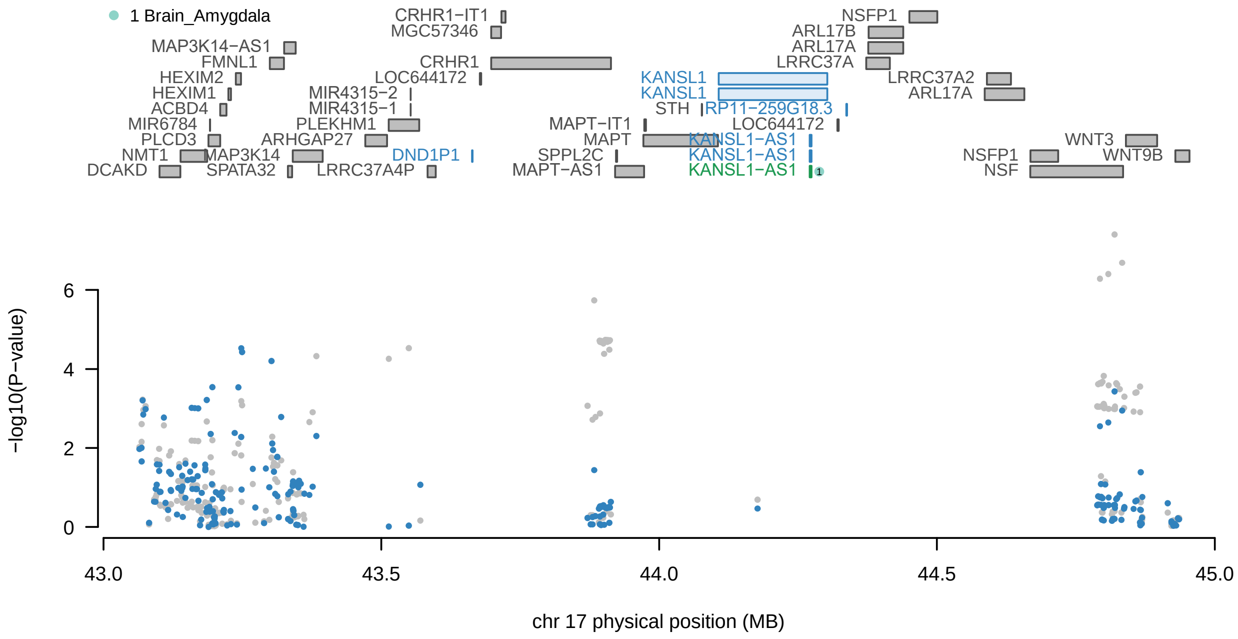

Supplementary Figure 9o. Regional Association Plot for Locus 16 from g-factor Conditional Analyses. The top half of the panel displays all of the genes located in the locus window. Genes that were marginally significant are highlighted in blue while those that were jointly significant are highlighted in green. The color and numbering of the point next to jointly significant green genes indicates the specific tissue type (legend in upper-left) for which that gene was jointly significant. The bottom half depicts the Manhattan plot of the GWAS SNP effects within the locus prior to (grey) and after (blue) conditioning on the jointly significant genes in green. There were no marginally significant genome-wide effects within this locus after conditioning on the predicted expression of the KANSL1-AS1 gene in the amygdala. Dots are vertically positioned along the y-axis of the Manhattan plot according to their two-sided -log10(p-values).

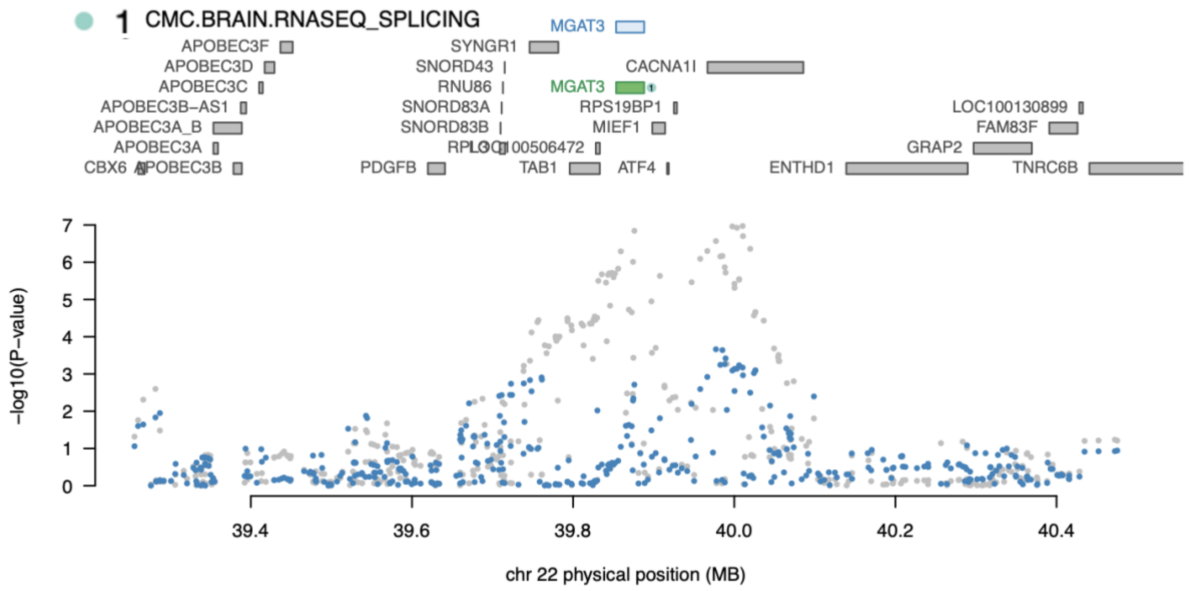

Supplementary Figure 9p. Regional Association Plot for Locus 17 from g-factor Conditional Analyses. The top half of the panel displays all of the genes located in the locus window. Genes that were marginally significant are highlighted in blue while those that were jointly significant are highlighted in green. The color and numbering of the point next to jointly significant green genes indicates the specific tissue type (legend in upper-left) for which that gene was jointly significant. The bottom half depicts the Manhattan plot of the GWAS SNP effects within the locus prior to (grey) and after (blue) conditioning on the jointly significant genes in green. There were no marginally significant genome-wide effects within this locus after conditioning on the predicted expression of the MGAT3 gene in the Common Mind Consortium RNA-seq dlPFC tissue. Dots are vertically positioned along the y-axis of the Manhattan plot according to their two-sided -log10(p-values).

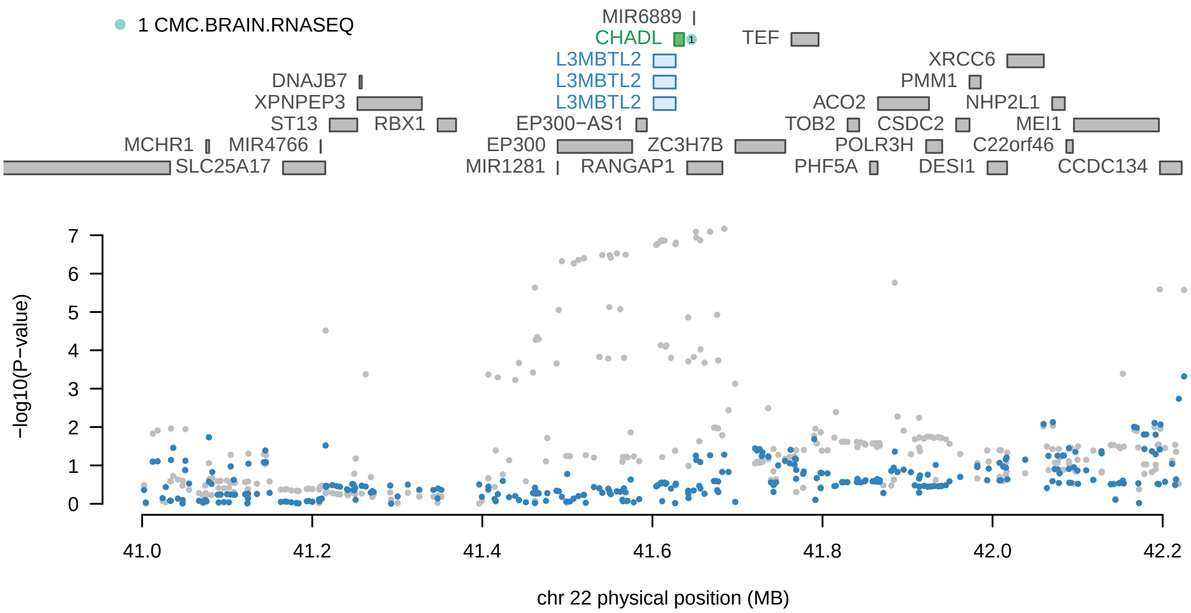

Supplementary Figure 9q. Regional Association Plot for Locus 18 from g-factor Conditional Analyses. The top half of the panel displays all of the genes located in the locus window. Genes that were marginally significant are highlighted in blue while those that were jointly significant are highlighted in green. The color and numbering of the point next to jointly significant green genes indicates the specific tissue type (legend in upper-left) for which that gene was jointly significant. The bottom half depicts the Manhattan plot of the GWAS SNP effects within the locus prior to (grey) and after (blue) conditioning on the jointly significant genes in green. There were no marginally significant genome-wide effects within this locus after conditioning on the predicted expression of the CHADL gene in the Common Mind Consortium RNA-seq dlPFC tissue. Dots are vertically positioned along the y-axis of the Manhattan plot according to their two-sided -log10(p-values).

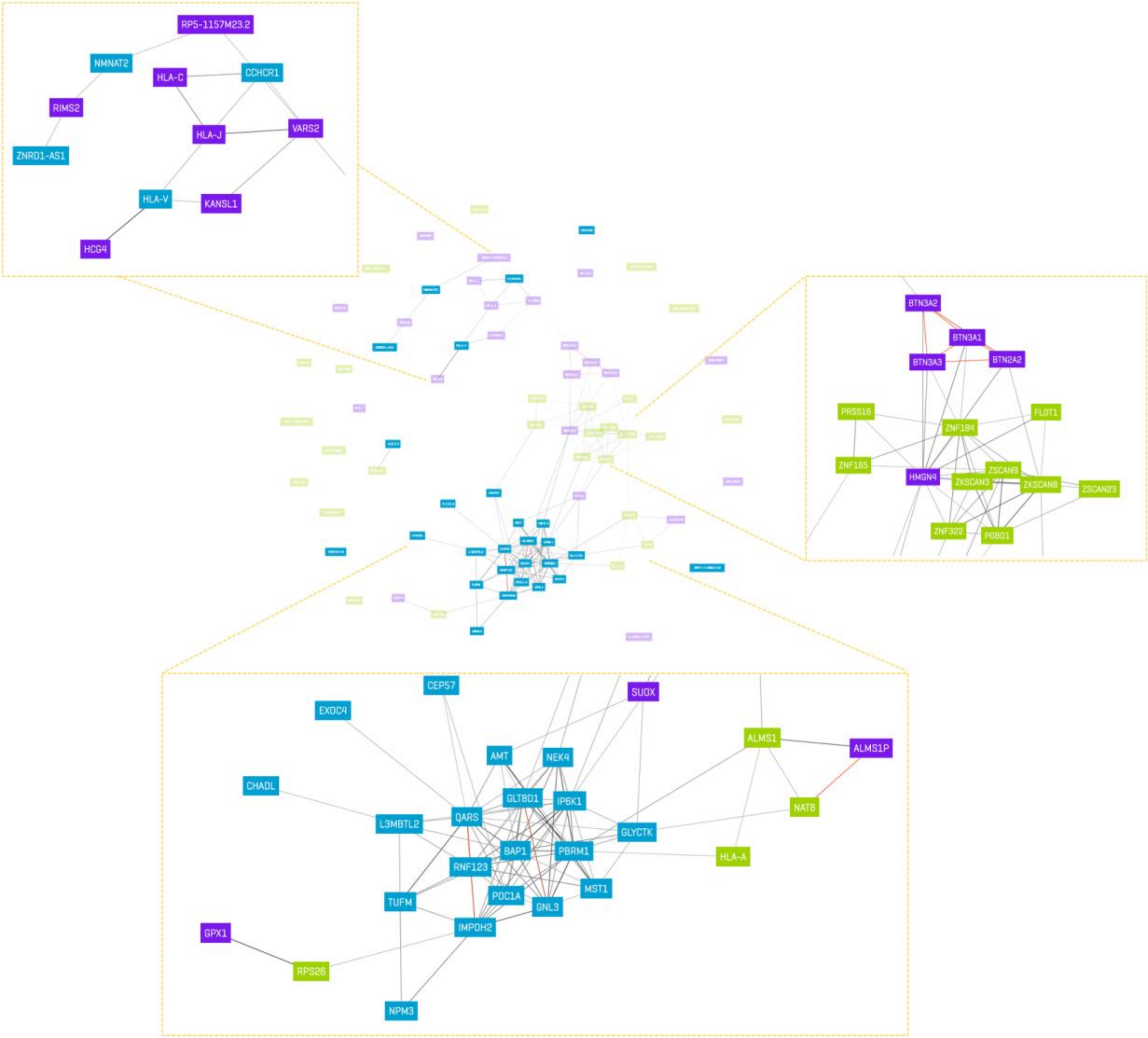

Supplementary Figure 10. g-factor Gene Co-expression Network. Figure depicts gene co-expression network for g-factor created using *Gene Network v2.0* for N=31,499 public RNA sequencing samples. Genes used as input to create the co-expression network included those genes that were significant at a Bonferroni corrected threshold for 52,849 tests and did not overlap with significant Q_{Gene} hits for the same gene and tissue type. These genes were restricted still further to those unique gene IDs across tissue types for a total of 79 genes used as input. As 3 of these genes were not in the gene network database, the plot above was constructed using a total of 76 gene IDs as input. Gene cluster 1 is depicted in blue (27 total genes). Gene cluster 2 is depicted in green (27 total genes). Gene cluster 3 is depicted in purple (22 total genes). Darker lines indicate stronger co-expression, with red lines depicted negative patterns of co-expression. Due to the size of the network, particular clusters of genes are depicted in greater detail in the yellow dashed boxes.

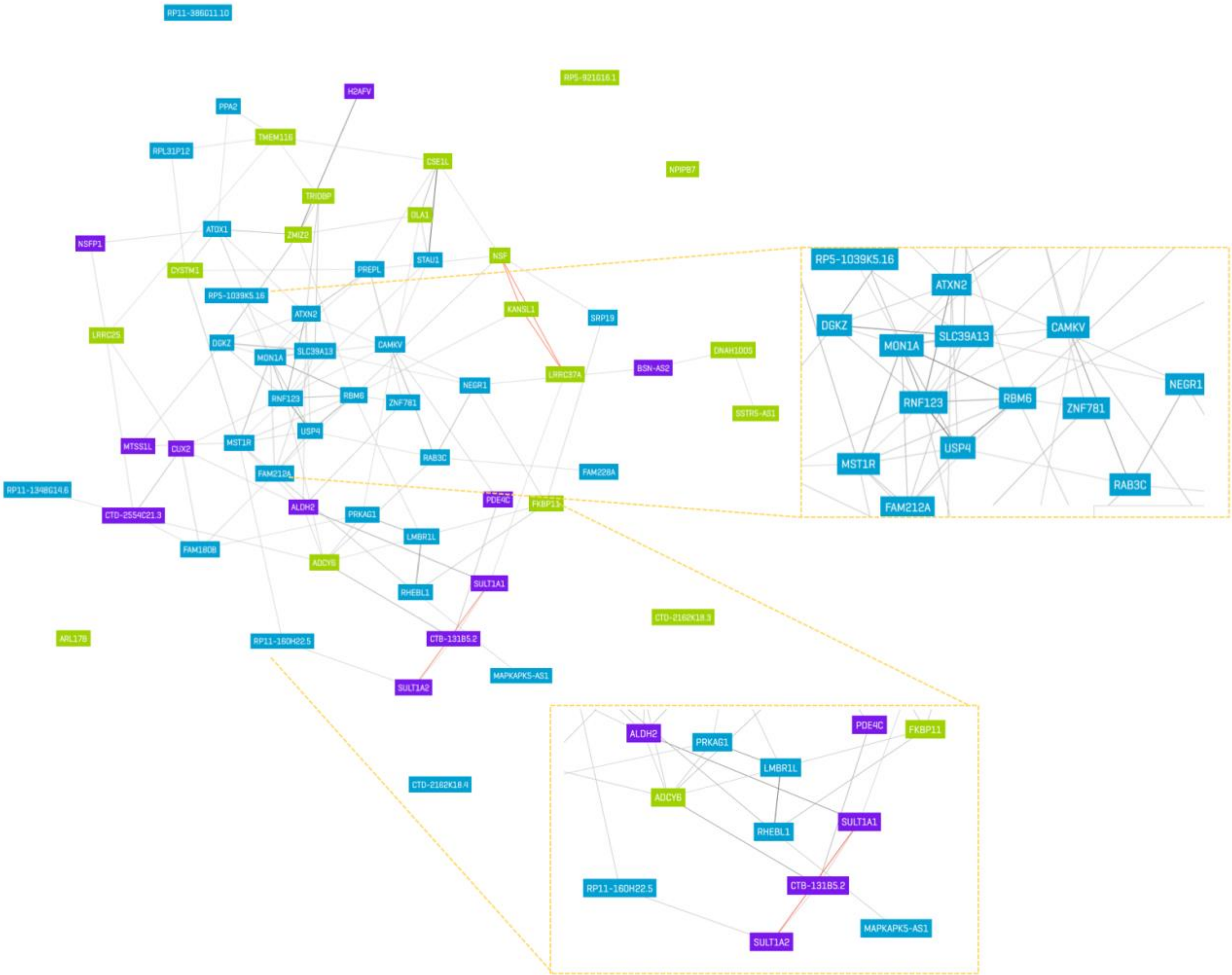

Supplementary Figure 11. Q_{Gene} Gene Co-expression Network. Figure depicts gene co-expression network for Q_{Gene} created using *Gene Network v2.0* for N=31,499 public RNA sequencing samples. Genes used as input to create the co-expression network included the 62 unique gene IDs that were significant at a Bonferroni corrected threshold for 52,849 tests. As 3 of these genes were not in the gene network database, the plot above was constructed using a total of 59 gene IDs as input. Gene cluster 1 is depicted in blue (30 total genes). Gene cluster 2 is depicted in green (18 total genes). Gene cluster 3 is depicted in purple (11 total genes). Darker lines indicate stronger co-expression, with red lines depicted negative patterns of co-expression. Due to the size of the network, particular clusters of genes are depicted in greater detail in the yellow dashed boxes.

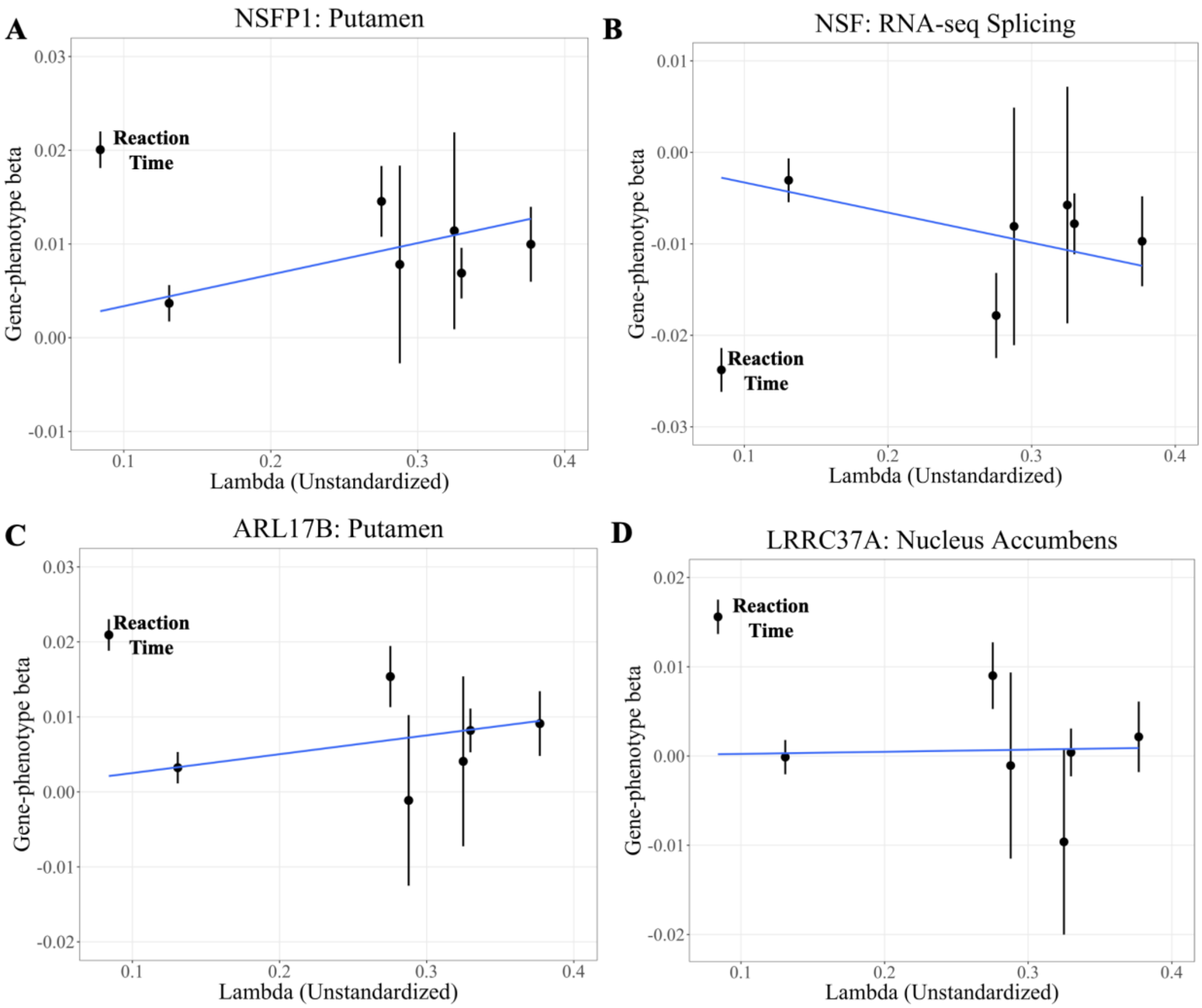

Supplementary Figure 12. Scatterplot of 17q21.31 Q_{Gene} hits. Scatter plot of Gene-phenotype regression coefficients (betas) estimated from FUSION against unstandardized genetic factor loadings form common factor model for genomic *g* estimated using LDSC genetic covariance matrix as input. Scatter plots are depicted for the 4 unique Q_{Gene} hits for the most significant tissue in the 17q21.31 region. Panel A depicts the NSFP1 gene in the putamen panel. Panel B depicts the NSF gene in the RNA-seq splicing panel. Panel C depicts the ARL17B gene in the putamen panel. Panel D depicts the LRRC37A gene in the nucleus accumbens panel. The solid blue line reflects the linear regression line based on all seven data points with the intercepts fixed to 0 to reflect the expectation from a common pathways model that the gene-phenotype regression relationship is 0 for an indicator that loads on the factor at 0. The data point for reaction time is highlighted across panels as it deviated the most strongly from this regression line. For all panels, the sample sizes for the cognitive variables is: reaction time (*n* = 330,024); matrix pattern recognition (*n* = 11,356); verbal numerical reasoning (*n* = 171,304); symbol digit substitution (*n* = 87,741); memory pairs-matching test (*n* = 331,679); tower rearranging (*n* = 11,263), trail making test-B (*n* = 78,457). Error bars reflect +/- 1 standard error of the betas in all panels.

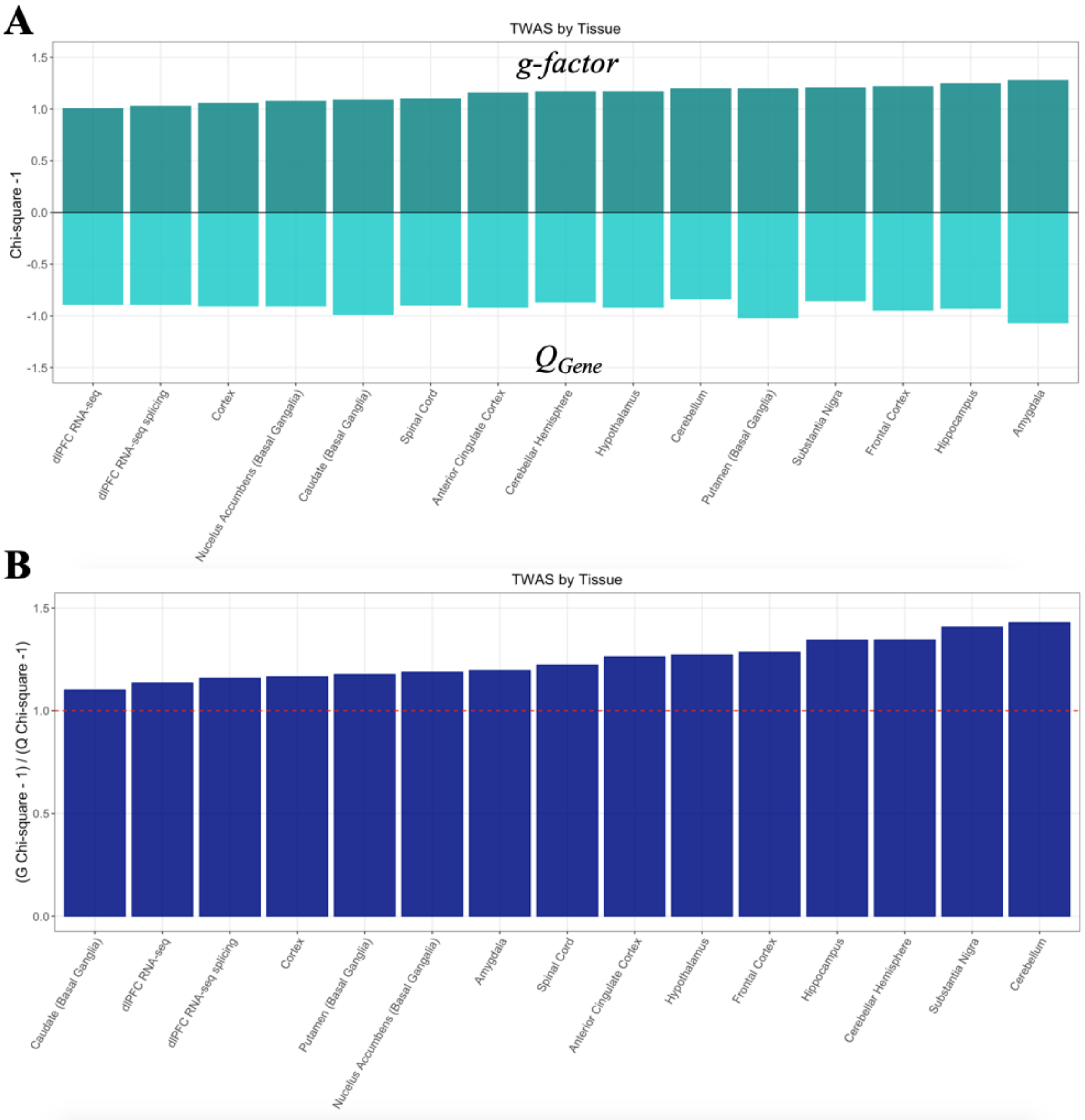

Supplementary Figure 13. Mean chi-square across Tissues. Panel A depicts the for the g-factor on the top half and Q_{Gene}*-1 on the bottom for the mean $\chi^2 - 1$. Bars are depicted in ascending order of the average values for the g-factor. Panel B depicts the ratio of mean $\chi^2 - 1$ for the g-factor over Q_{Gene} and are again depicted in ascending order for this particular ratio. A red dashed line is depicted at 1, as this would indicate equal signal for Q_{Gene} and the *g*-factor. For both panels, Q_{Gene} is scaled to a 1 degree of freedom χ^2 test statistic for comparative purposes, and 1 is subtracted from all χ^2 averages given a χ^2 null of 1.

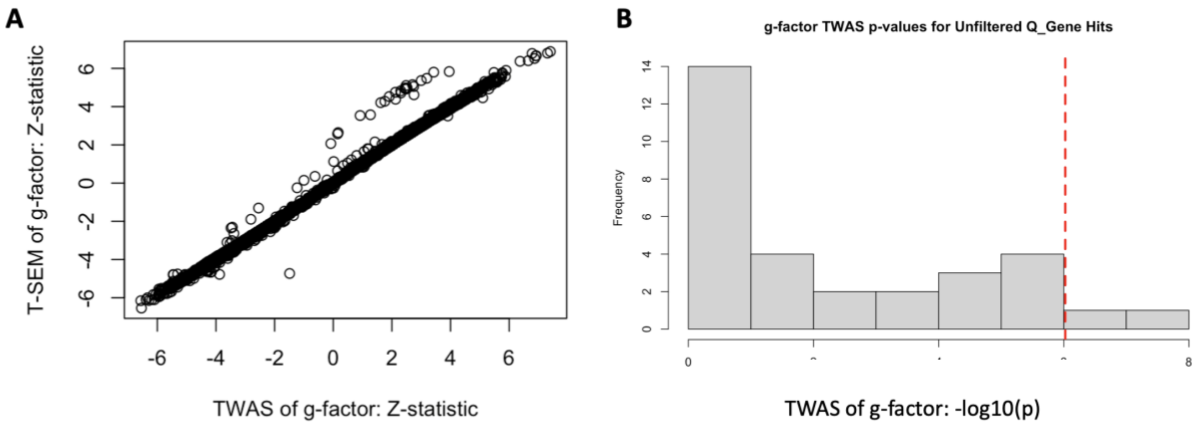

Supplementary Figure 14. TWAS vs T-SEM of *g*-factor. ***Panel A*** depicts the scatterplot of the Z-statistics for the TWAS of the *g*-factor GWAS summary statistics on the x-axis against T-SEM of the *g*-factor on the y-axis. In line with simulation results, we observe a strong concordance across these two estimates. ***Panel B*** depicts the histogram of the two-sided, -log10(*p*) values for TWAS Z-statistics for the 23 genes identified as significant for Q_{Gene} that were not filtered out based on Q_{SNP}. The red-dashed line is shown at the Bonferroni corrected significance threshold. The 2 values to the right of this line are then likely to be false positives for *g* given the significant heterogeneity in gene expression across the seven cognitive indicators identified by T-SEM.

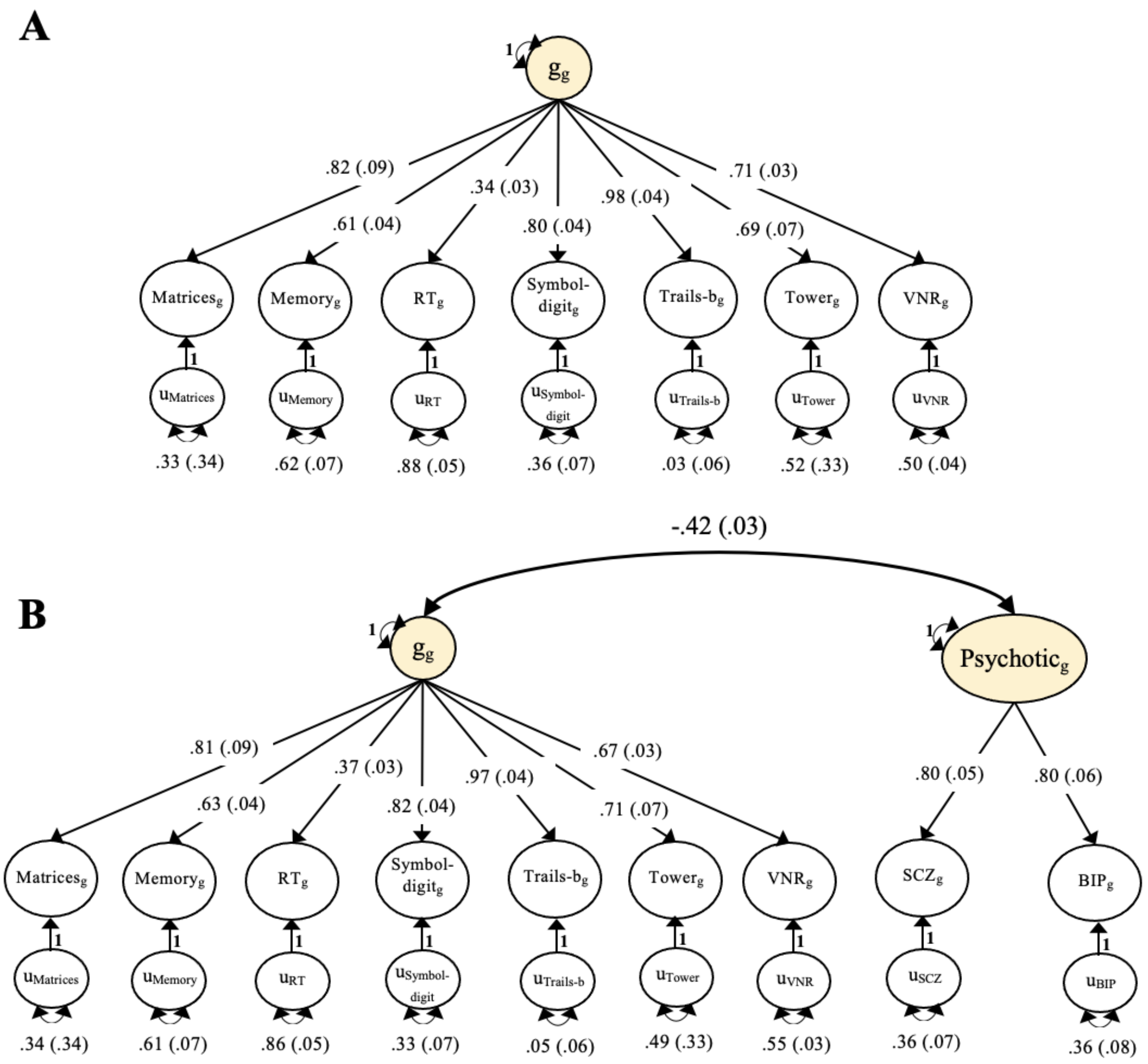

Supplementary Figure 15. Factor Models estimated using Genome-wide, Zero-order Matrix. *Panel A:* Standardized results for g-factor model fit to the genome-wide S-LDSC matrix. This reflects the baseline model used to estimate enrichment of genetic variance of the g-factor, where all factor loadings are fixed from this model in Step 1 and the indicator uniquenesses (i.e., residual variances) and factor variance are re-estimated within each functional annotation. The factor loadings will differ very slightly from those presented in the original description of genomic g[1], as those estimates were obtained from a common factor model fit to a matrix estimated using LDSC (as opposed to S-LDSC). *Panel B:* Standardized results for correlated factors model between a g-factor and psychotic disorders factor fit to S-LDSC matrix for the annotation including all SNPs. This model was used to examine enrichment of the genetic correlation between these two factors. For identification purposes, the factor loadings of the psychotic disorders are set to equality across schizophrenia and bipolar disorder. Indicators in both panels are presented as circles to reflect the fact that these are unobserved heritability estimates from S-LDSC. Note that the genetic correlation across *g* and the psychotic disorders factor is just slightly different than the genetic correlation reported in the main text for results examining mediation of genetic overlap via tissue-specific gene expression. This is due to different genetic covariances matrices being used as input, where tissue-specific gene expression results utilize the genome-wide matrix estimated from LDSC and results presented here use the genome-wide matrix esimated using *S*-LDSC.

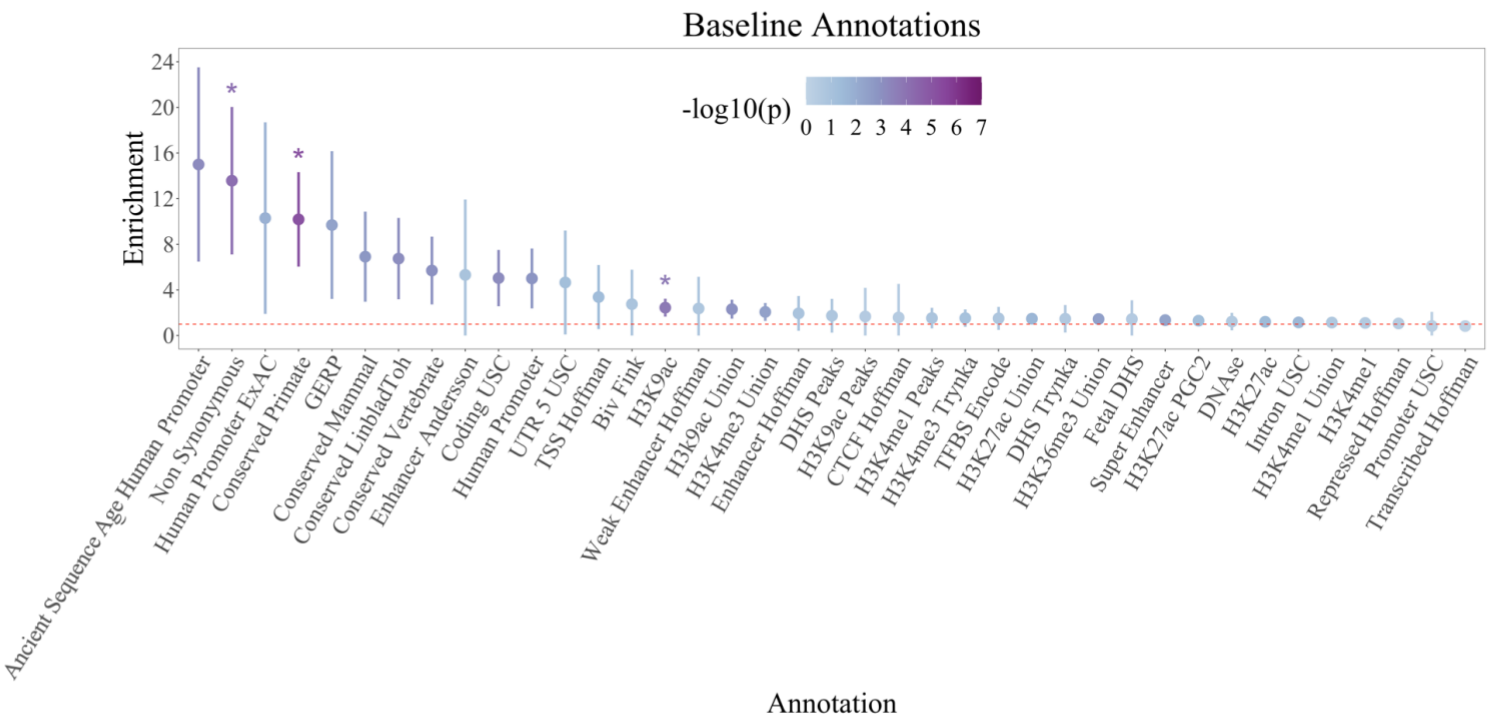

Supplementary Figure 16a. Enrichment of Baseline Annotations for g-factor. Dots are depicted in descending order based on the point estimate for enrichment of the *g*-factor. Dots are shaded according to the significance of the enrichment estimate using a one-sided -log10(p)-value. Dots that were significant at a Bonferroni corrected threshold for 155 tests are depicted with a *. The red dashed line reflects the null (enrichment = 1). The dots depict the enrichment point estimates. Error bars depict 95% CIs. The scaling of the y-axis across enrichment graphs differs due to discrepant ranges in CIs across annotations.

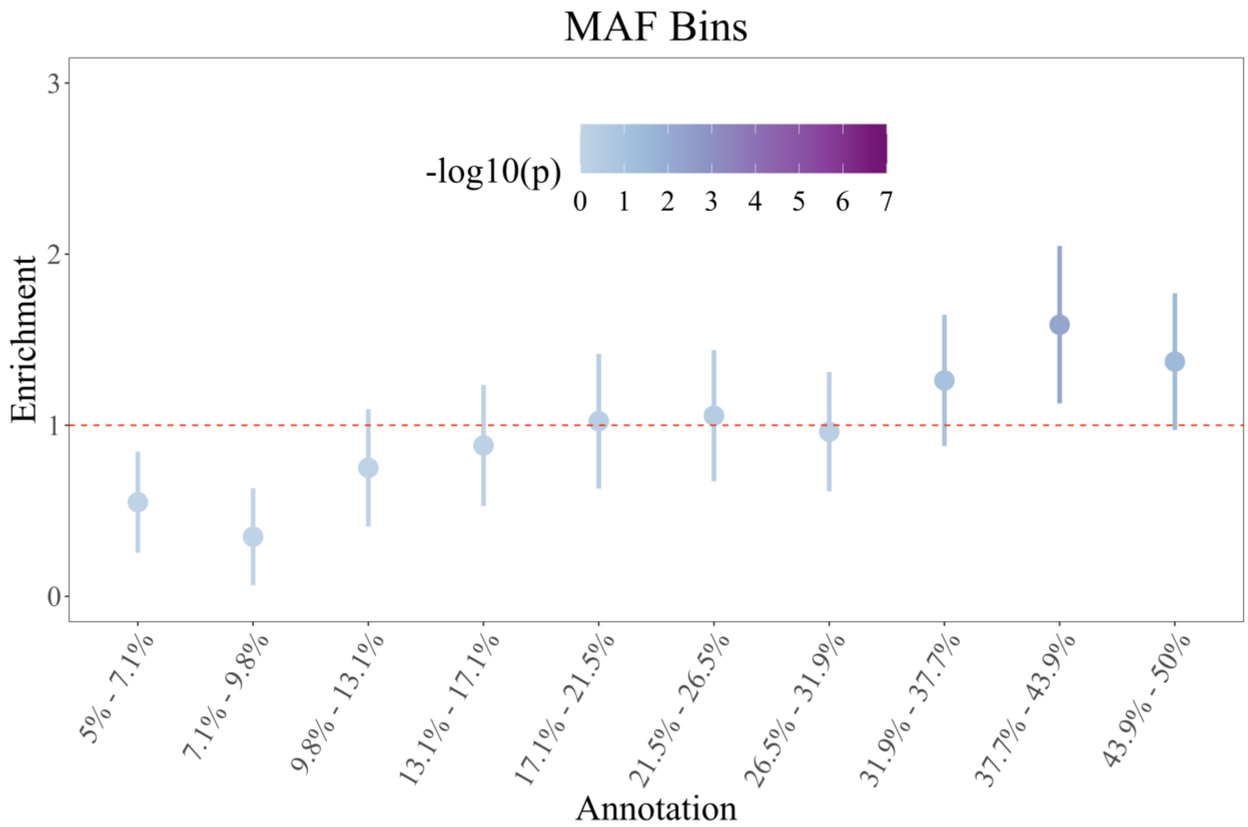

Supplementary Figure 16b. Enrichment of MAF Annotations for g-factor. Dots are depicted in order of the minor allele frequency bins. Dots are shaded according to the significance of the enrichment estimate using a one-sided -log10(p)-value. No MAF bins were significant at a Bonferroni corrected threshold for 155 tests. The red dashed line reflects the null (enrichment = 1). The dots depict the enrichment point estimates. Error bars depict 95% CIs. The scaling of the y-axis across enrichment graphs differs due to discrepant ranges in CIs across annotations.

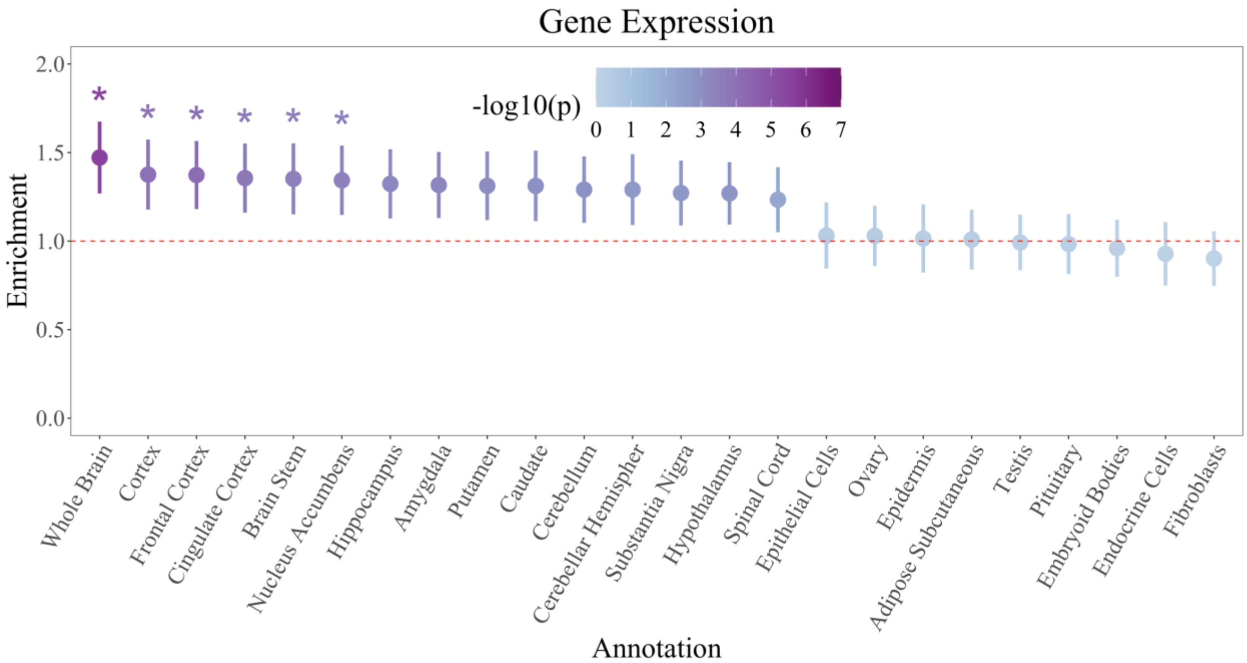

Supplementary Figure 16c. Enrichment of Gene Expression Annotations for g-factor. Dots are depicted in descending order based on the point estimate for enrichment of the *g*-factor. Dots are shaded according to the significance of the enrichment estimate using a one-sided -log10(p)-value. Dots that were significant at a Bonferroni corrected threshold for 155 tests are depicted with a *. The red dashed line reflects the null (enrichment = 1). The dots depict the enrichment point estimates. Error bars depict 95% CIs. The scaling of the y-axis across enrichment graphs differs due to discrepant ranges in CIs across annotations.

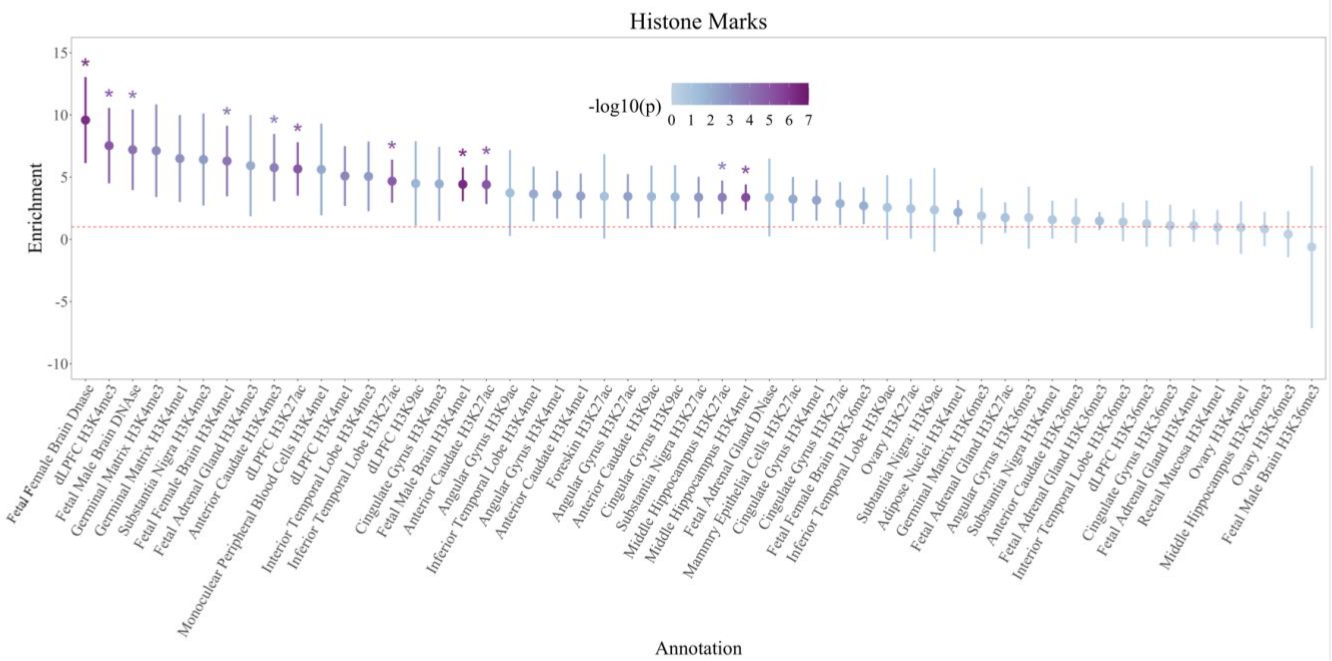

Supplementary Figure 16d. Enrichment of Histone Mark Annotations for g-factor. Dots are depicted in descending order based on the point estimate for enrichment of the *g*-factor. Dots are shaded according to the significance of the enrichment estimate using a one-sided -log10(p)-value. Dots that were significant at a Bonferroni corrected threshold for 155 tests are depicted with a *. The red dashed line reflects the null (enrichment = 1). The dots depict the enrichment point estimates. Error bars depict 95% CIs. The scaling of the y-axis across enrichment graphs differs due to discrepant ranges in CIs across annotations.

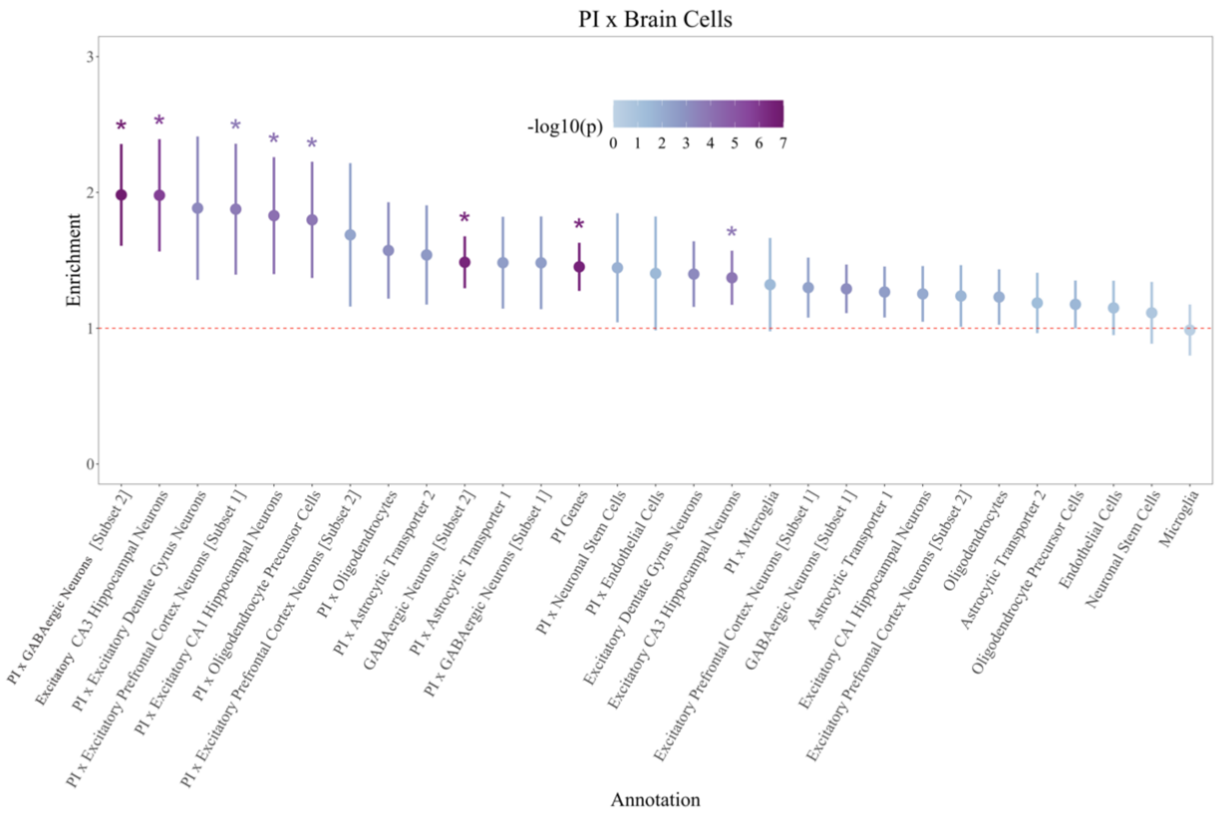

Supplementary Figure 16e. Enrichment of PI x Brain Cell Annotations for g-factor. Dots are depicted in descending order based on the point estimate for enrichment of the *g*-factor. Dots are shaded according to the significance of the enrichment estimate using a one-sided -log10(p)-value. Dots that were significant at a Bonferroni corrected threshold for 155 tests are depicted with a *. The red dashed line reflects the null (enrichment = 1), the dots depict enrichment point estimates, the error bars depict 95% CIs, and dots that were significant at a Bonferroni corrected threshold for 155 tests are depicted with a *.

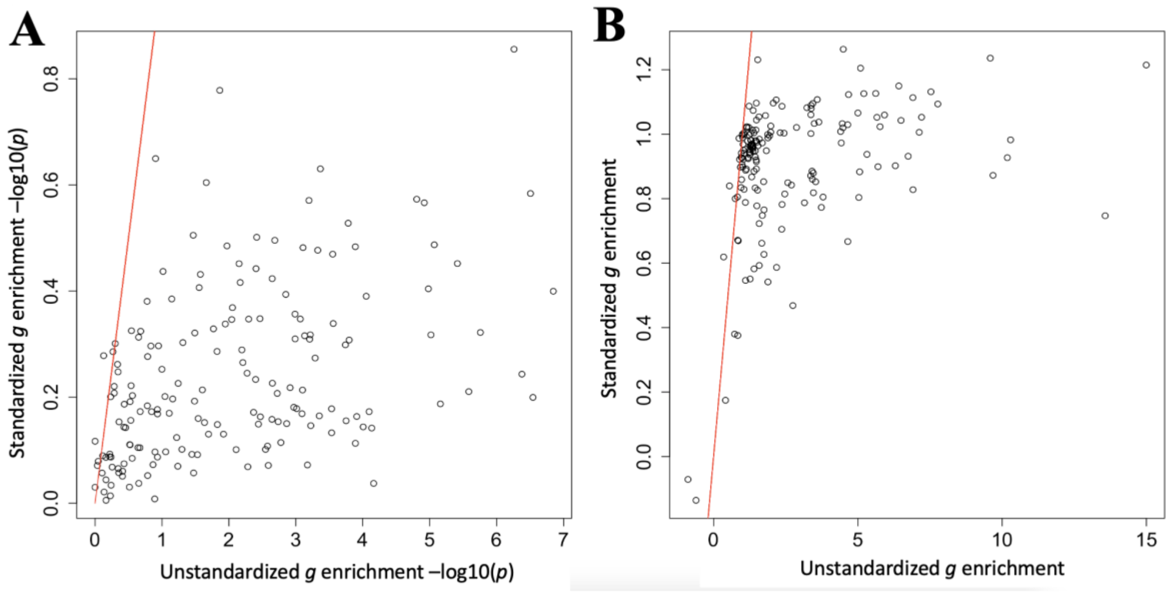

Supplementary Figure 17. Scatter plots for *g* enrichment fit to stratified covariance and correlation matrices. *Panel A* depicts the relationship between the two-sided -log10 p-values for *g* fit to stratified covariance matrices (unstandardized) on the x-axis and to stratified correlation matrices (standardized) on the y-axis. *Panel B* depicts the same scatter plot but with enrichment point estimates on both axes. Red lines reflect the unstandardized g enrichment estimates predicting itself in both panels, with dots below the line reflecting more significant estimates for unstandardized *g* relative to standardized *g*.

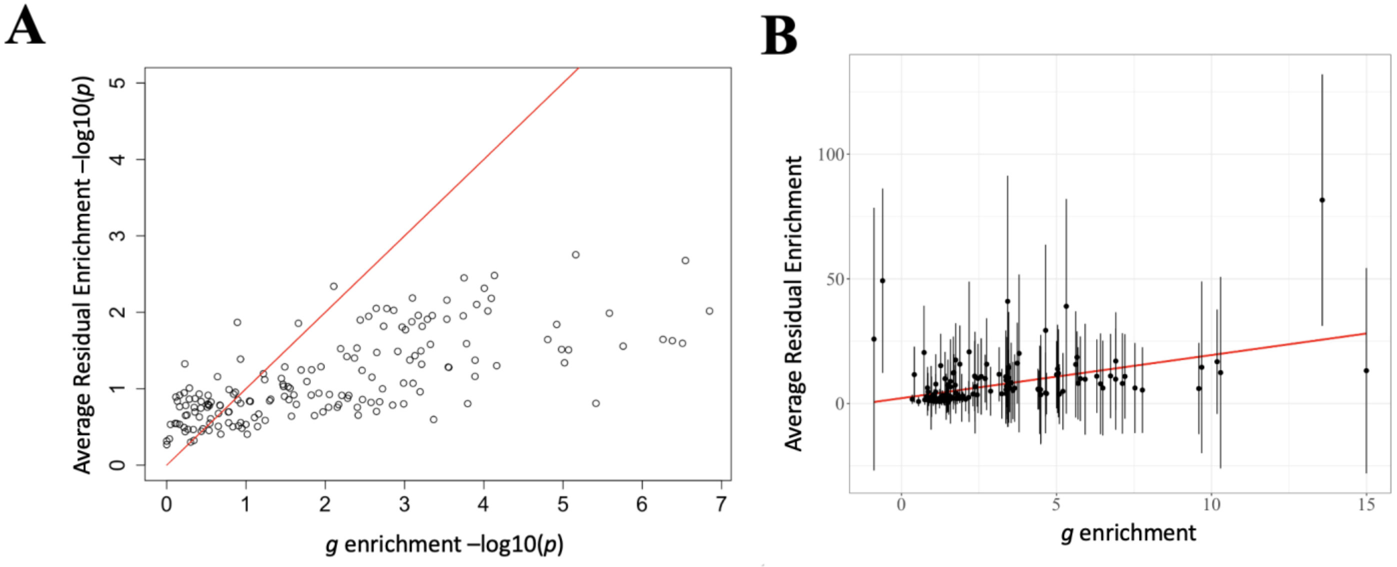

Supplementary Figure 18. Scatterplots of residual and genetic *g* enrichment. *Panel A* depicts the relationship between the two-sided -log10 p-values for *g* on the x-axis and the average of the -log10 p-values across the residuals for the seven cognitive indicators on the y-axis. *Panel B* depicts the same scatter plot but with enrichment point estimates on both axes. That is, the placement of the dots on the x-axis reflects the enrichment point estimate for *g* and the average residual enrichment on the y-axis. Error bars reflect +/- 1 SE for the residual point estimates. Red lines reflect the g enrichment estimates predicting itself in both panels, with dots below the line reflecting more significant estimates for g relative to the residuals.

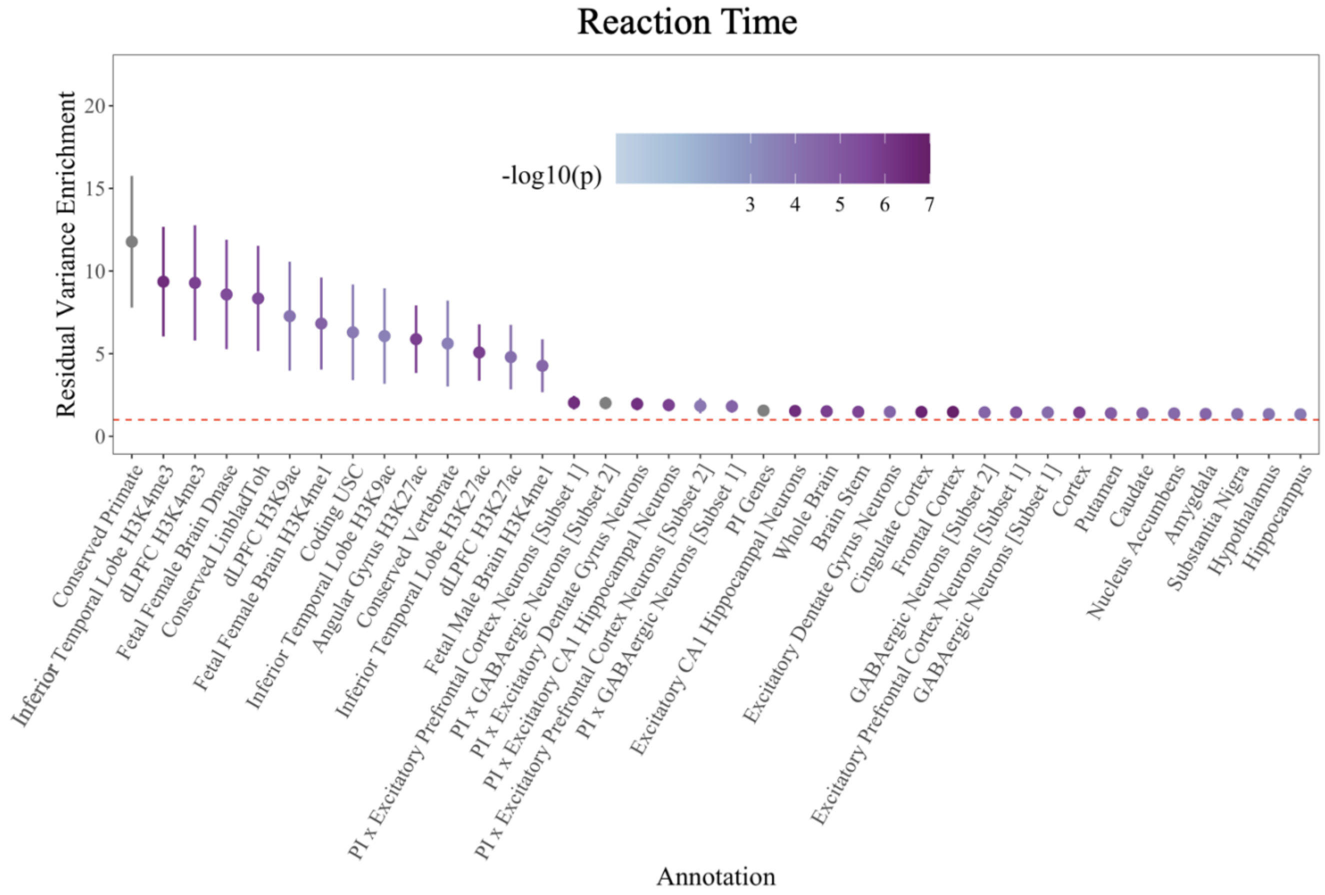

Supplementary Figure 19a. Enrichment for Residual Variance in Reaction Time. Dots are depicted in descending order based on the point estimate for enrichment of the residual variance in reaction time. Dots are shaded according to the significance of the enrichment estimate using a one-sided -log10(p)-value. Figure depicts the 38 significant estimates for reaction time using a Bonferroni corrected threshold for 155 tests. The ed dashed line reflects the null (enrichment = 1), the dots depict the enrichment point estimates, and the error bars depict 95% CIs.

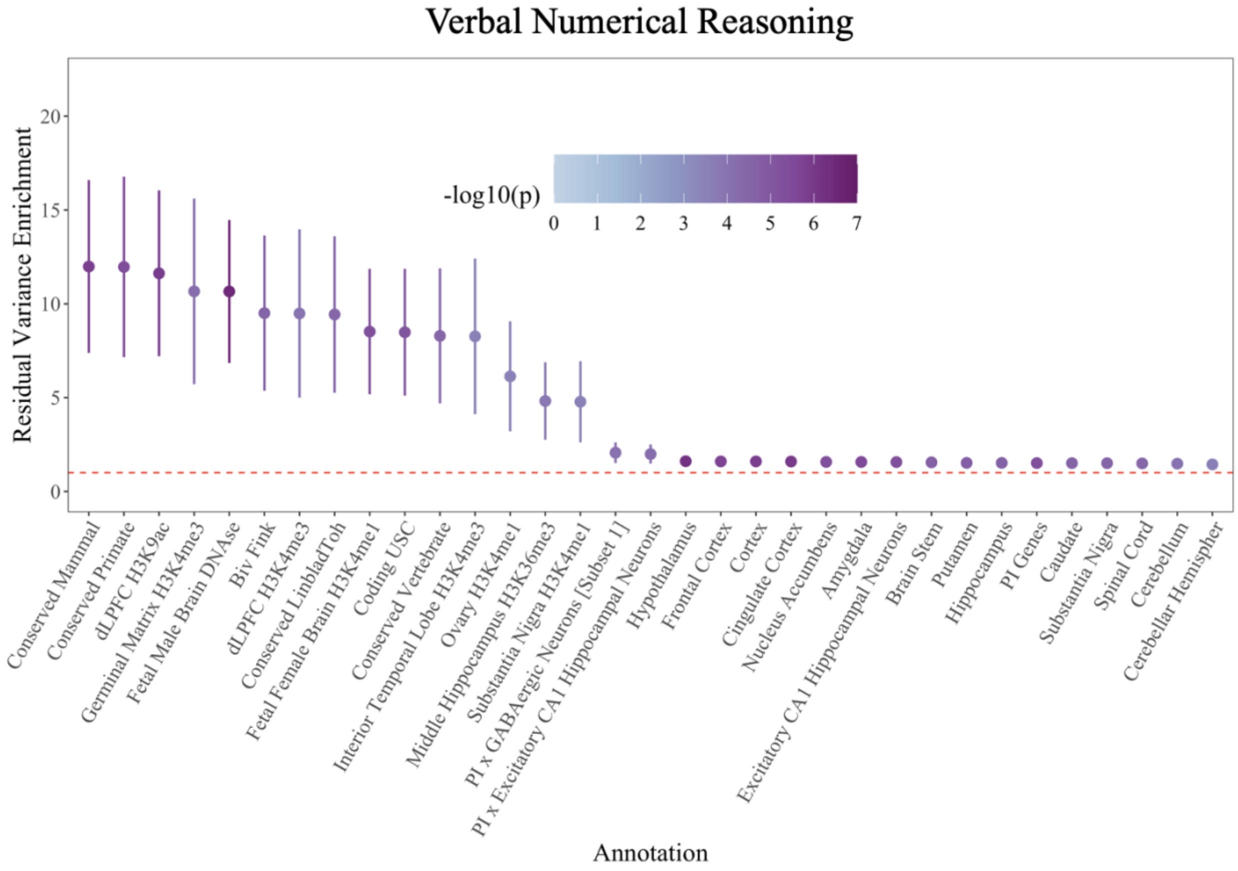

Supplementary Figure 19b. Enrichment for Residual Variance in Verbal Numerical Reasoning. Dots are depicted in descending order based on the point estimate for enrichment of the residual variance in verbal numerical reasoning. Dots are shaded according to the significance of the enrichment estimate using a one-sided -log10(p)-value. Figure depicts the 33 significant estimates using a Bonferroni corrected threshold for 155 tests for verbal numerical reasoning. The red dashed line reflects the null (enrichment = 1), the dots depict the enrichment point estimates, and the error bars depict 95% CIs.

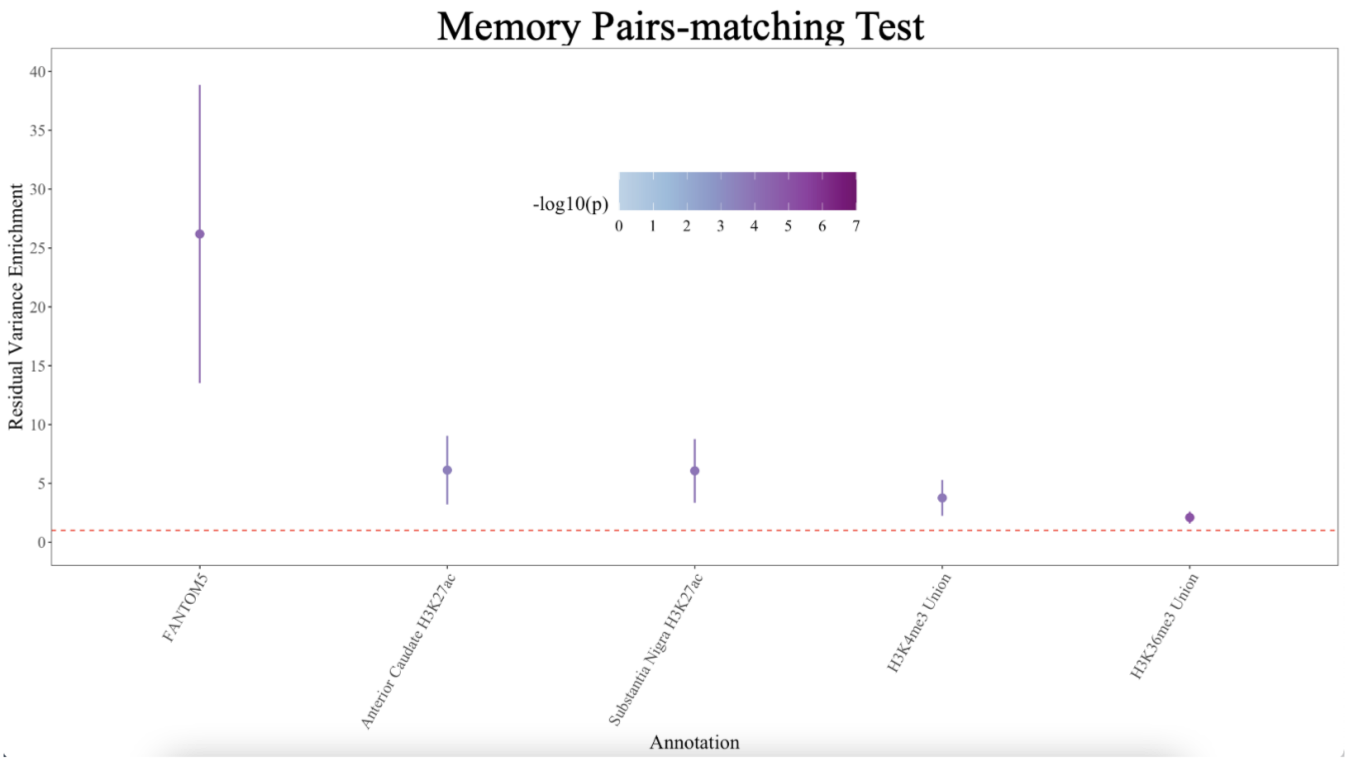

Supplementary Figure 19c. Enrichment for Residual Variance in the Memory Pairs-matching Test. Dots are depicted in descending order based on the point estimate for enrichment of the residual variance in the memory pairs-matching test. Dots are shaded according to the significance of the enrichment estimate using a one-sided -log10(p)-value. Figure depicts the 5 significant estimates at a Bonferroni corrected threshold for memory pairs-matching test. The red dashed line reflects the null (enrichment = 1), the dots depict the enrichment point estimates, and the error bars depict 95% CIs.

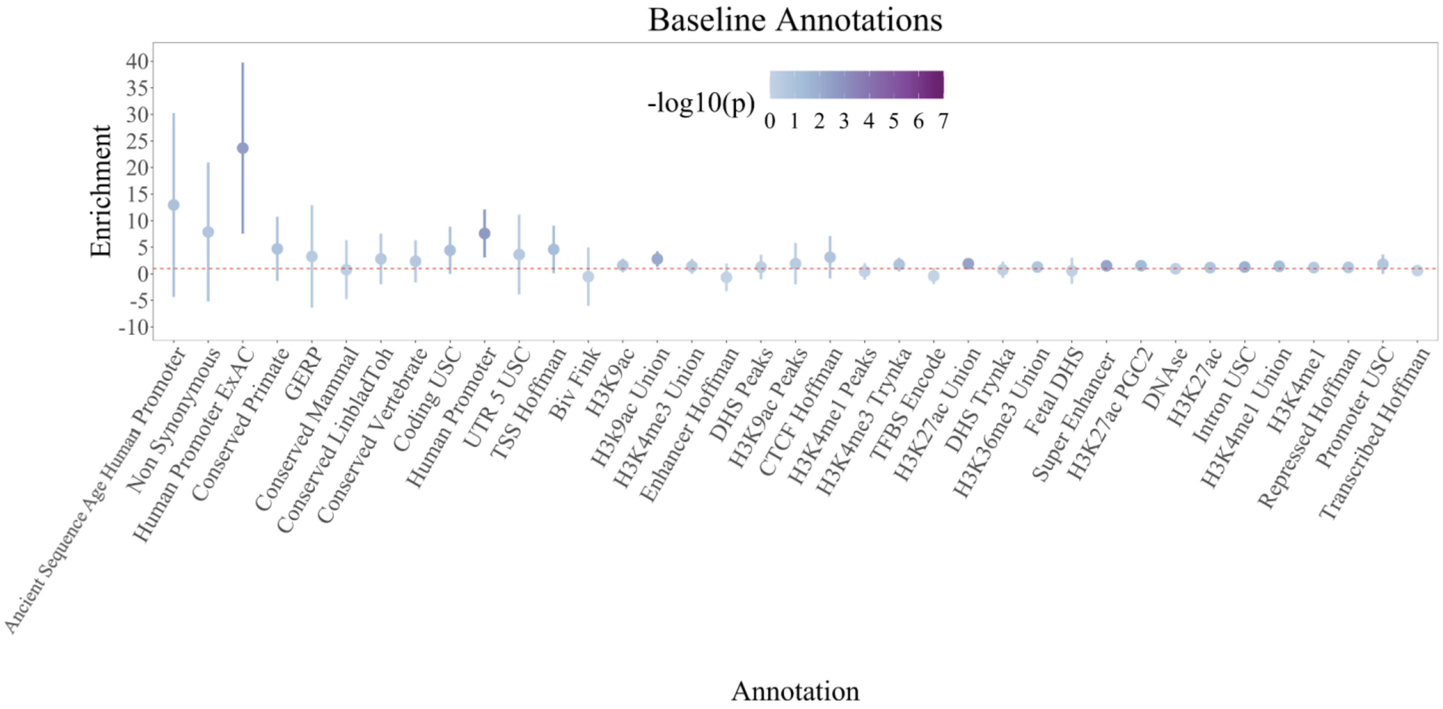

Supplementary Figure 20a. Enrichment of Baseline Annotations for covariance between the *g*-factor and psychotic disorders factor. For comparative purposes, dots are depicted in descending order based on the point estimate for enrichment of the *g*-factor as per Figure S6a. Dots are shaded according to the significance of the enrichment estimate using a one-sided -log10(p)-value. No baseline annotations were significant at a Bonferroni corrected threshold for 155 tests. The red dashed line reflects the null (enrichment = 1), the dots depict the enrichment point estimates, and the error bars depict 95% CIs. The scaling of the y-axis differs across enrichment graphs due to widely discrepant ranges in point estimates and CIs across annotations.

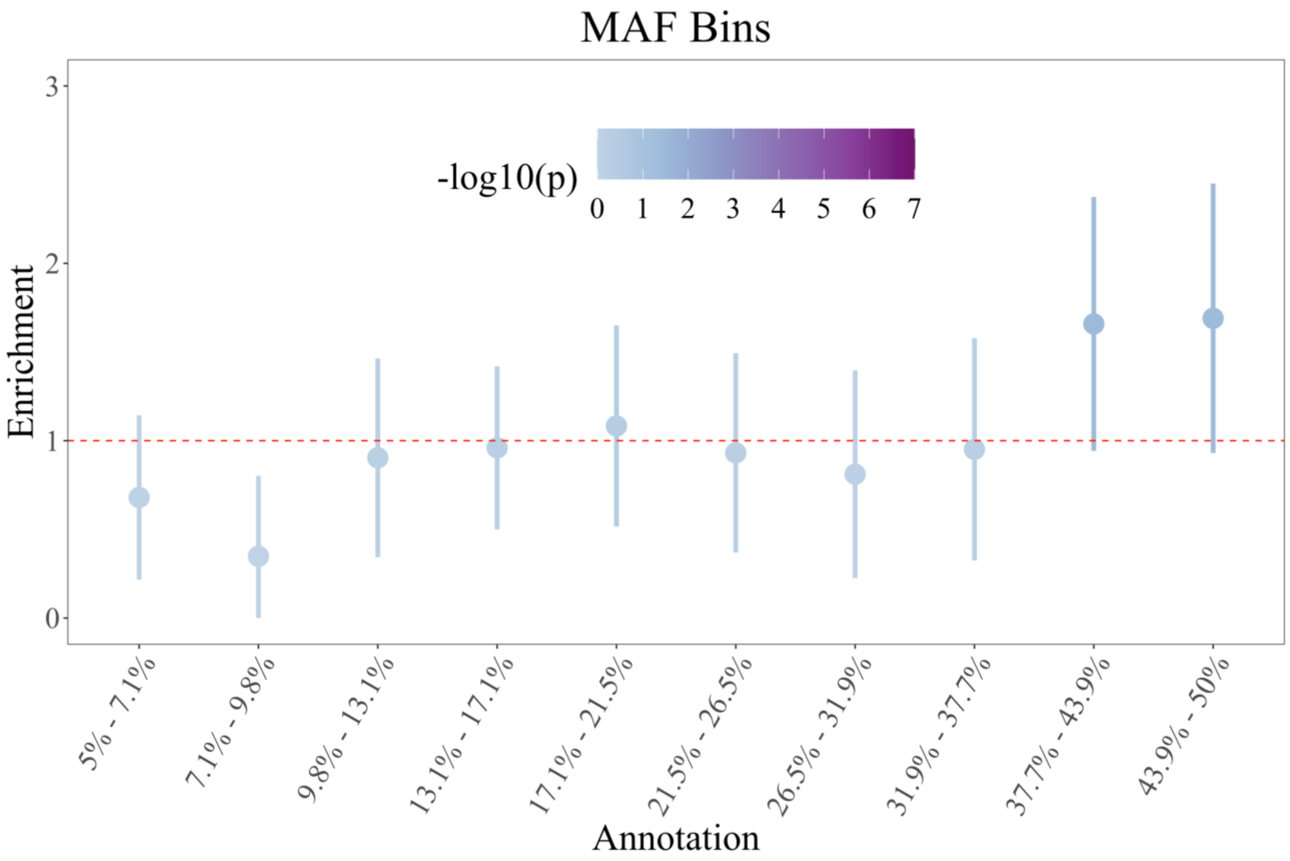

Supplementary Figure 20b. Enrichment of MAF Annotations for covariance between the *g*-factor and psychotic disorders factor. Dots are depicted in order of the minor allele frequency bins. Dots are shaded according to the significance of the enrichment estimate using a one-sided -log10(p)-value. No MAF bins were significant at a Bonferroni corrected threshold for 155 tests. The red dashed line reflects the null (enrichment = 1), the dots depict the enrichment point estimates, and the error bars depict 95% CIs. The scaling of the y-axis across enrichment graphs differs due to discrepant ranges in CIs across annotations.

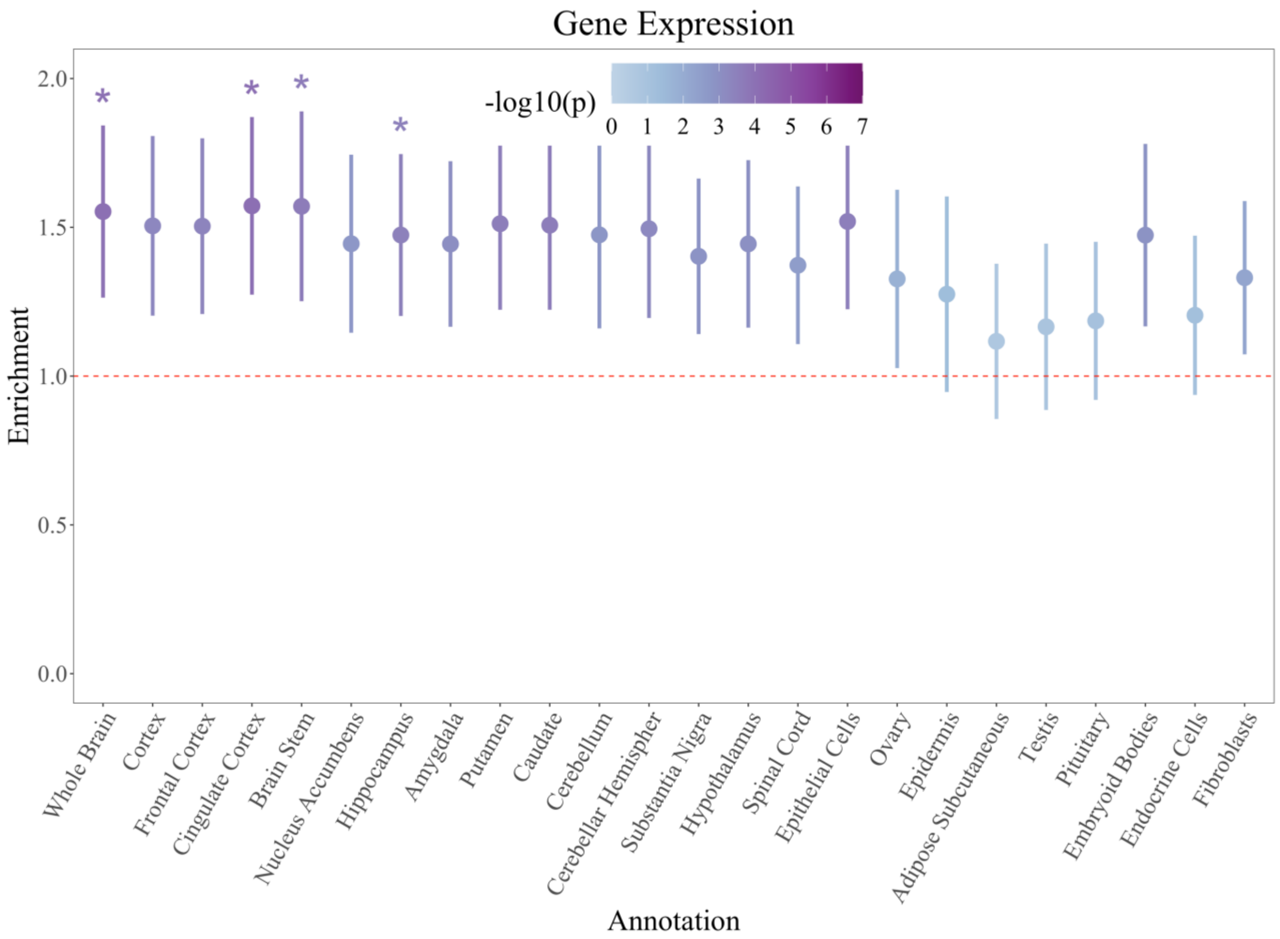

Supplementary Figure 20c. Enrichment of Gene Expression Annotations for covariance between the *g*-factor and psychotic disorders factor. For comparative purposes, dots are depicted in descending order based on the point estimate for enrichment of the *g*-factor as per Figure S7c. Dots are shaded according to the significance of the enrichment estimate using a one-sided -log10(p)-value. Dots that were significant at a Bonferroni corrected threshold for 155 tests are depicted with a *. The red dashed line reflects the null (enrichment = 1), the dots depict the enrichment point estimates, and the error bars depict 95% CIs. The scaling of the y-axis across enrichment graphs differs due to discrepant ranges in CIs across annotations.

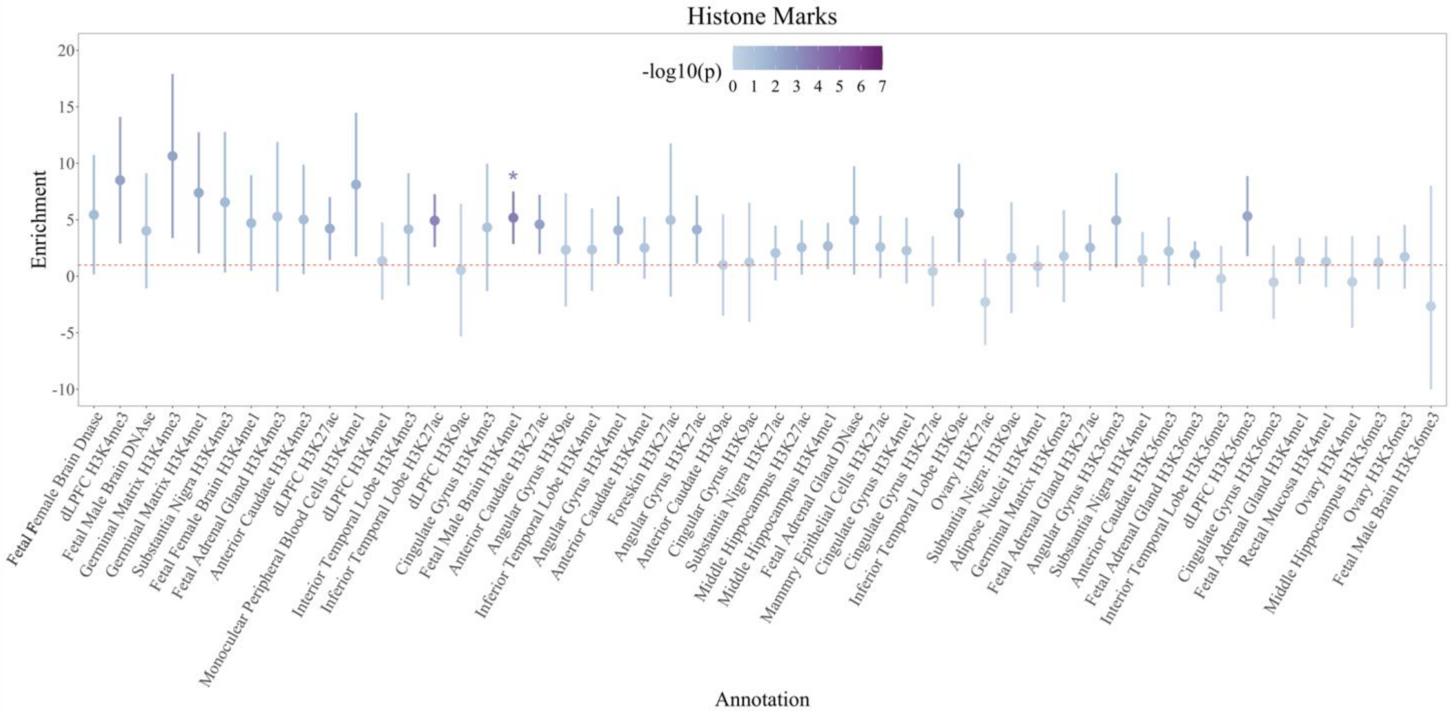

Supplementary Figure 20d. Enrichment of Histone Mark Annotations for covariance between the *g*-factor and psychotic disorders factor. For comparative purposes, dots are depicted in descending order based on the point estimate for enrichment of the *g*-factor as per Figure S7d. Dots are shaded according to the significance of the enrichment estimate using a one-sided -log10(p)-value. Dots that were significant at a Bonferroni corrected threshold for 155 tests are depicted with a *. The red dashed line reflects the null (enrichment = 1), the dots depict the enrichment point estimates, and the error bars depict 95% CIs. The scaling of the y-axis across enrichment graphs differs due to discrepant ranges in CIs across annotations.

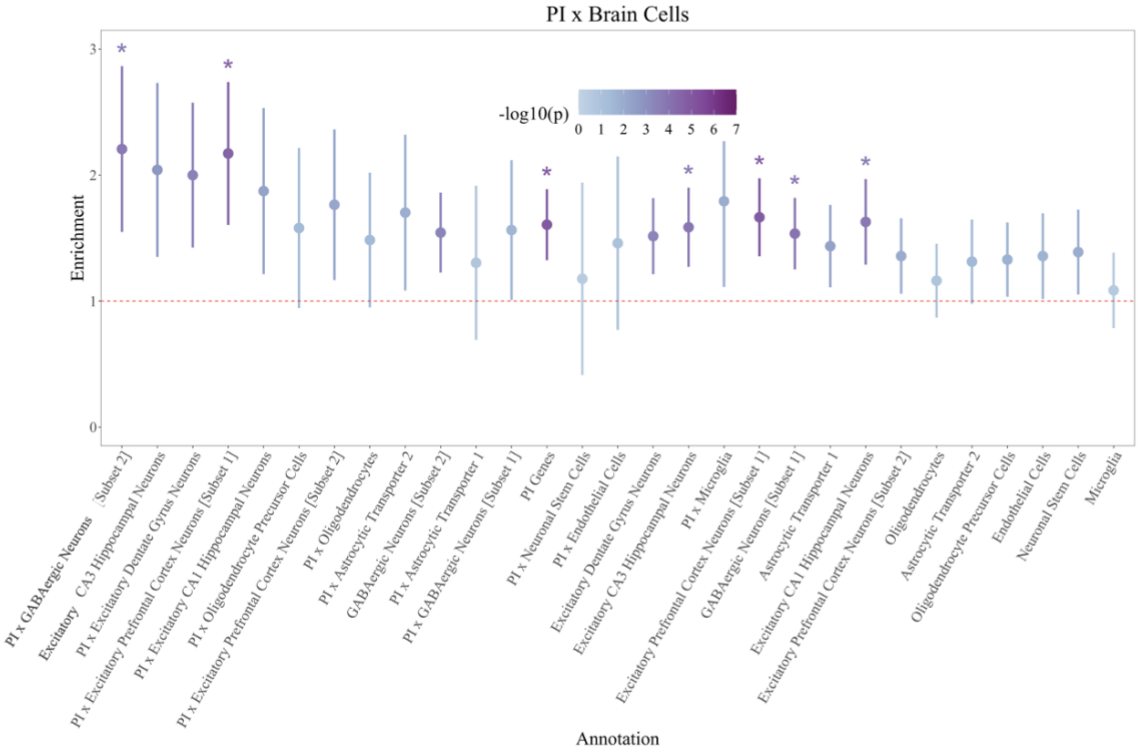

Supplementary Figure 20e. Enrichment of PI x Brain Cell Annotations for covariance between the *g*-factor and psychotic disorders factor. For comparative purposes, dots are depicted in descending order based on the point estimate for enrichment of the *g*-factor as per Figure S7e. Dots are shaded according to the significance of the enrichment estimate using a one-sided -log10(p)-value. Dots that were significant at a Bonferroni corrected threshold for 155 tests are depicted with a *. The red dashed line reflects the null (enrichment = 1), the dots depict the enrichment point estimates, and the error bars depict 95% CIs.
