# Supplementary material for: Transcriptome-wide and stratified genomic structural equation modeling identify neurobiological pathways shared across diverse cognitive traits
Source: Nat Commun. 2022 Oct 21;13:6280. doi: 10.1038/s41467-022-33724-9 (PMC9586980; doi:10.1038/s41467-022-33724-9)
Supplement: Supplementary file 3 — Description of Additional Supplementary Files [file 41467_2022_33724_MOESM3_ESM.pdf]

Description of Additional Supplementary Files

File Name: Supplementary Data 1
Description: Summary of Cognitive Phenotypes

File Name: Supplementary Data 2
Description: T-SEM Simulation Results

File Name: Supplementary Data 3
Description: TWAS versus T-SEM Simulation Results

File Name: Supplementary Data 4
Description: 218 Multivariate TWAS hits for g-factor

File Name: Supplementary Data 5
Description: Results for Conditional Analysis

File Name: Supplementary Data 6
Description: Gene-set Analyses

File Name: Supplementary Data 7
Description: Summary of External Phenotypes

File Name: Supplementary Data 8
Description: Mediation of Factor Correlations by Gene Expression

File Name: Supplementary Data 9
Description: 156 Qgene hits

File Name: Supplementary Data 10
Description: TWAS summary data by tissue type

File Name: Supplementary Data 11
Description: Multivariate Genetic Enrichment (Stratified Genomic SEM)
