# Supplementary material for: Transcriptome-wide and stratified genomic structural equation modeling identify neurobiological pathways shared across diverse cognitive traits
Source: Nat Commun. 2022 Oct 21;13:6280. doi: 10.1038/s41467-022-33724-9 (PMC9586980; doi:10.1038/s41467-022-33724-9)
Supplement: Supplementary file 5 — Reporting Summary [file 41467_2022_33724_MOESM5_ESM.pdf]

Corresponding author(s): Andrew Grotzinger

Last updated by author(s): Sep 22, 2022

Reporting Summary

Nature Portfolio wishes to improve the reproducibility of the work that we publish. This form provides structure for consistency and transparency in reporting. For further information on Nature Portfolio policies, see our Editorial Policies and the Editorial Policy Checklist.

Statistics

For all statistical analyses, confirm that the following items are present in the figure legend, table legend, main text, or Methods section.

n/a	Confirmed	
☐	☒	The exact sample size (*n*) for each experimental group/condition, given as a discrete number and unit of measurement
☐	☒	A statement on whether measurements were taken from distinct samples or whether the same sample was measured repeatedly
☐	☒	The statistical test(s) used AND whether they are one- or two-sided<br>*Only common tests should be described solely by name; describe more complex techniques in the Methods section.*
☒	☐	A description of all covariates tested
☐	☒	A description of any assumptions or corrections, such as tests of normality and adjustment for multiple comparisons
☐	☒	A full description of the statistical parameters including central tendency (e.g. means) or other basic estimates (e.g. regression coefficient) AND variation (e.g. standard deviation) or associated estimates of uncertainty (e.g. confidence intervals)
☐	☒	For null hypothesis testing, the test statistic (e.g. *F*, *t*, *r*) with confidence intervals, effect sizes, degrees of freedom and *P* value noted<br>*Give P values as exact values whenever suitable.*
☒	☐	For Bayesian analysis, information on the choice of priors and Markov chain Monte Carlo settings
☒	☐	For hierarchical and complex designs, identification of the appropriate level for tests and full reporting of outcomes
☐	☒	Estimates of effect sizes (e.g. Cohen's *d*, Pearson's *r*), indicating how they were calculated

Our web collection on statistics for biologists contains articles on many of the points above.

Software and code

Policy information about availability of computer code

Data collection	No software was used for data collection.
Data analysis	Genetic correlations, enrichment analyses estimated using Stratified Genomic sEM, and Transcriptome-wide SEM were conducted using the Genomic SEM package available for download at https://github.com/GenomicSEM/GenomicSEM. This includes a help page and publicly available code for T-SEM, which is introduced and validated in the current submission. Genomic SEM v0.0.5 was implemented using R version 3.5.1 and Univariate TWAS was estimated using FUSION, which was downloaded on August 13th, 2020 from: http://gusevlab.org/projects/fusion/

For manuscripts utilizing custom algorithms or software that are central to the research but not yet described in published literature, software must be made available to editors and reviewers. We strongly encourage code deposition in a community repository (e.g. GitHub). See the Nature Portfolio guidelines for submitting code & software for further information.

Data

Policy information about availability of data

All manuscripts must include a data availability statement. This statement should provide the following information, where applicable:

- Accession codes, unique identifiers, or web links for publicly available datasets
- A description of any restrictions on data availability
- For clinical datasets or third party data, please ensure that the statement adheres to our policy

The data that support the findings of this study are all publicly available or can be requested for access. Specific download links for various datasets are directly below.
Summary statistics for the g-factor and the seven, individual cognitive traits are available from:

https://datashare.is.ed.ac.uk/handle/10283/3756
Summary statistics for bipolar disorder data can be found here:
https://figshare.com/articles/dataset/PGC3_bipolar_disorder_GWAS_summary_statistics/14102594
Summary statistics for schizophrenia can be found here:
https://figshare.com/articles/dataset/scz2022/19426775
Summary statistics for major depressive disorder can be found here:
https://datashare.ed.ac.uk/handle/10283/3203
Summary statistics for anxiety can be downloaded here:
https://drive.google.com/drive/folders/1fguHvz7l2G45sbMI9h_veQun4aXNTy1v
Summary statistics for Alzheimer's disease can be easily requested here:
https://www.niagads.org/datasets/ng00075
Summary statistics for Parkinson's disease can be downloaded here:
https://drive.google.com/drive/folders/10bGj6HfAXgl-JslpI9ZJIL_JIgZyktxn
Data from gnomAD used to identify PI genes for creation of annotations can be downloaded here: https://storage.googleapis.com/gnomad-public/release/2.1.1/constraint/gnomad.v2.1.1.lof_metrics.by_gene.txt.bgz
Gene count data per cell for creation of annotations were obtained from: https://storage.googleapis.com/gtex_additional_datasets/single_cell_data/GTEx_droncseq_hip_pcf.tar
Data which maps individual cells to cell types (e.g. neuron, astrocyte etc.) were obtained from: https://static-content.springer.com/esm/art%3A10.1038%2Fnmeth.4407/MediaObjects/41592_2017_BFnmeth4407_MOESM10_ESM.xlsx
Links to the LD-scores, reference panel data, and the code used to produce the current results can all be found at: https://github.com/GenomicSEM/GenomicSEM/wiki
Links to the BaselineLD v2.2 annotations can be found here:
https://data.broadinstitute.org/alkesgroup/LDSCORE/
Links to the reference weights used for FUSION from GTEx and CMC can be found here:
http://gusevlab.org/projects/fusion/

Field-specific reporting

Please select the one below that is the best fit for your research. If you are not sure, read the appropriate sections before making your selection.

☐ Life sciences ☒ Behavioural & social sciences ☐ Ecological, evolutionary & environmental sciences

For a reference copy of the document with all sections, see nature.com/documents/nr-reporting-summary-flat.pdf

Behavioural & social sciences study design

All studies must disclose on these points even when the disclosure is negative.

Study description	The current study develops and validates a novel method--Transcriptome-wide Structural Equation Modeling (T-SEM)--for examining the association of tissue-specific gene expression within a multivariate system of human complex traits. We apply T-SEM to examine the effect of tissue-specific gene expression on a diverse set of seven cognitive traits. In doing so, we identify subsets of genes that are broadly relevant to cognitive function as separable from genes with highly specific effects on certain cognitive domains. We go on to apply another recently developed method, Stratified Genomic SEM, to examine multivariate functional enrichment of these same cognitive domains.
Research sample	We utilize publicly available GWAS summary statistics from UK Biobank for the seven cognitive traits and from the Psychiatric Genomics Consortium (PGC) for the external psychiatric correlates. The collateral gene expression and functional annotation data that are used as input to T-SEM and Stratified Genomic SEM, respectively, are also available for download. As Genomic SEM relies on ld-score regression (LDSC) to construct genetic covariance matrices, and LDSC requires summary statistics to be within a single ethnic population due to differences in linkage disequilibrium across populations, we use only summary statistics restricted to European populations. Therefore, our findings are not representative beyond European populations, and as we highlight in the manuscript it will be critical for future work to re-examine these research questions for different ancestry groups.
Sampling strategy	As we use previously collected GWAS data from outside groups, and did not collect any participant samples ourselves, this is not applicable.
Data collection	This is not applicable as we use previously collected GWAS data that we were not involved in collecting.
Timing	This is not applicable as we use previously collected data. We began curating GWAS summary statistics in May 2021 and put a freeze on including any new GWAS datasets in August 2021.
Data exclusions	We use only summary statistics from European populations due to the requirements of LDSC, as noted above.
Non-participation	This is not applicable as we use previously collected data.
Randomization	This is not applicable as we use previously collected data that we were not involved in collecting. We can say that the original GWAS

Randomization	study designs were not randomized in the sense that participants were either psychiatric cases or controls in the GWAS, and there were no further conditions to randomize participants into.

Reporting for specific materials, systems and methods

We require information from authors about some types of materials, experimental systems and methods used in many studies. Here, indicate whether each material, system or method listed is relevant to your study. If you are not sure if a list item applies to your research, read the appropriate section before selecting a response.

Materials & experimental systems

n/a	Involved in the study
☒	☐ Antibodies
☒	☐ Eukaryotic cell lines
☒	☐ Palaeontology and archaeology
☒	☐ Animals and other organisms
☒	☐ Human research participants
☒	☐ Clinical data
☒	☐ Dual use research of concern

Methods

n/a	Involved in the study
☒	☐ ChIP-seq
☒	☐ Flow cytometry
☒	☐ MRI-based neuroimaging
